# Tumour Burden Reporting in Phase III Clinical Trials of Metastatic Lung, Breast, and Colorectal Cancers: A Systematic Review

**DOI:** 10.3390/cancers14133262

**Published:** 2022-07-03

**Authors:** Mariachiara Santorsola, Vincenzo Di Lauro, Guglielmo Nasti, Michele Caraglia, Maurizio Capuozzo, Francesco Perri, Marco Cascella, Gabriella Misso, Alessandro Ottaiano

**Affiliations:** 1 Istituto Nazionale Tumori di Napoli, IRCCS “G. Pascale”, Via M. Semmola, 80131 Naples, Italy; mariachiara.santorsola@istitutotumori.na.it (M.S.); dilaurovincenzo87@gmail.com (V.D.L.); g.nasti@istitutotumori.na.it (G.N.); f.perri@istitutotumori.na.it (F.P.); m.cascella@istitutotumori.na.it (M.C.); 2 Department of Precision Medicine, University of Campania “L. Vanvitelli”, Via L. De Crecchio 7, 80138 Naples, Italy; michele.caraglia@unicampania.it (M.C.); gabriella.misso@unicampania.it (G.M.); 3 Department of Pharmacy, ASL-Naples-3, 80056 Ercolano, Italy; capuozzo.mauri@libero.it

**Keywords:** tumour burden, phase III studies, non-small-cell lung cancer, breast cancer, colorectal cancer

## Abstract

**Simple Summary:**

The initial tumour burden is a strong and well-known prognostic factor in oncology. A systematic review was performed to examine if and how the initial tumour burden is reported in phase III clinical trials in the most frequent and deadly cancers. Seventy trials were selected, which mostly included biologic agents. The identification of low-burden metastatic disease was performed in 28.6% of studies; it was a stratification factor for randomisation in only 25.7% of studies. In two studies, a significant imbalance between arms in patients with low-burden disease was revealed. Our findings emphasise the need for the better assessment of tumour burden in clinical trials.

**Abstract:**

Background: Randomised phase III clinical trials represent a methodological milestone to select effective drugs against metastatic cancers. In this context, and particularly in the efficacy assessment of biologic drugs, the initial metastatic tumour burden is a strong prognostic factor. Methods: A systematic literature review of randomised, phase III, first-line, clinical trials in metastatic breast, colorectal, and lung cancers, published from 2016 to 2021, was performed. Three groups of variables were collected: identity-, method- (including tumour burden assessment) and outcome-related. Results: Seventy trials were selected. A large portion of studies (41.4%) focused on the effects of biologic agents (signal inhibitors and immuno-therapies). A definition of low-burden disease based predominantly on the number of involved organs was reported in 28.6% of studies. No explicit reference to oligo-metastatic disease was found either in inclusion/exclusion criteria or in final descriptive data analyses. Disease extent, heterogeneously defined, was a stratification factor for randomisation in only 25.7% of studies. In two studies, a significant imbalance between arms in patients with low-burden disease was revealed. Conclusions: Attention to initial tumour burden in designing future clinical trials (including the harmonisation of definitions and the reporting of eventual oligo-metastatic disease, complete estimates of tumour volume, and its consideration as a stratification factor) should be increased.

## 1. Introduction

The evidence-based development of anti-cancer drugs relies on a clinical research plan going from phase I to III studies. In this view, randomised phase III studies represent the last step to demonstrate that an experimental drug has higher efficacy compared to a placebo or standard treatment. The inclusion criteria of patients in phase III studies are crucial to avoid the risk of biases potentially affecting the results. Stringent methodological rules (specific clinical characteristics, stratification factors, randomisation, etc.) are applied to guarantee the soundness and generalisability of final data [1,2]. The initial tumour burden of enrolled patients is a strong prognostic factor in solid tumours. For this reason, it is sometimes included as a stratification factor in the randomisation procedures of phase III comparative trials.

Furthermore, increasing insight into the definition and characterisation of tumour burden has highlighted the need to differentiate between poly- and oligo-metastatic diseases [3,4,5,6,7,8]. A crucial study [8] analysed and reorganised this issue with systematic appraisal. Experts and members of the European Society for Radiotherapy and Oncology (ESTRO) and European Organisation for Research and Treatment of Cancer (EORTC) made a consensus study to identify and classify oligometastatic diseases. The authors distinguished an “induced” oligo-metastatic disease (patients with history of poly-metastatic cancer before the diagnosis of oligo-metastatic disease) from a “genuine” oligo-metastatic disease (patients with no history of poly-metastatic disease before the diagnosis of oligo-metastatic disease). This definition translates into a strongly divergent clinical outcome for patients. Most importantly, the authors confirmed the “quantitative characteristics” of cancers as pivotal prognostic indicators in clinical research as well as crucial identifiers of the oligo-metastatic status (i.e., the number of involved organs and the number and size of lesions). From a pragmatic point of view, oligo-metastases can be identified as cancer involving no more than three lesions per organ with a maximum tumour size smaller than 7 cm [4,6,9] or a primary tumour (active or resected) with ≤5 metastatic masses easily resectable or controlled with local approaches [7].

Oligo-metastatic patients are an interesting model to study low-burden metastatic disease. They have a good prognosis since the disease has a long time course and is safely controlled with local treatments and less aggressive systemic therapies [5]. In fact, the median survival of oligo-metastatic colorectal cancer (omCRC) patients is generally better (about 44.0 months) compared to that of poly-metastatic disease patients (24.0 months) [10]. In this subset of patients, we previously documented specific genetic and immunologic characteristics [11], providing evidence that omCRC is a specific disease and not simply an evolutionary step towards more aggressive biological behaviour. Furthermore, we previously argued that hidden oligo-metastatic disease in phase III clinical trials could bias study results [12]. To this aim, a crucial methodological scientific question is raised: if and how the initial burden of metastatic disease, including the eventual oligo-metastatic status, is reported in phase III randomised clinical trials.

## 2. Materials and Methods

### 2.1. Search Strategy and Selection Criteria

A systematic review according to Preferred Reporting Item For Systematic Reviews and Meta-Analysis was performed [13]. Lung and female breast cancers are the most commonly diagnosed cancers worldwide (about 6 million new cases/year, 18% of new diagnoses of cancer), followed by colorectal (about 10% of new diagnoses) and prostate cancer (about 7%). However, while metastatic prostate cancer generally has an indolent course, metastatic lung, breast, and colorectal cancers are leading causes of cancer deaths with survivals, frequently lower than two years [14]. For this reason, we focused specifically on lung, breast, and colorectal cancers in which the initial burden of disease is more likely to influence the short-term prognosis in clinical trials. The search was conducted on March 2022 through PubMed and Google Scholar with a set of common key words (i.e., “therapy” AND “first line”) associated with variable key words depending on the type of cancer (breast: “breast cancer” OR “breast tumour”; colorectal cancer: “colorectal cancer” OR “colorectal tumour”; non-small cell lung cancer: “non-small cell lung cancer” OR “lung cancer”). The applied filters were “clinical trials” and “phase III studies”. In addition to key-word-based browsing, the analysis of the reference sections of the selected original papers was conducted to avoid the exclusion of further eligible articles. The following eligibility criteria for articles selection were applied: (1) phase III, prospective, comparative studies, (2) first-line therapy, (3) metastatic disease, (4) published from January 2016 to December 2021, (5) time-to-outcome (progression-free (PFS) and/or survival (OS)) as primary end-point, (6) reporting HRs (hazard ratios) for time-to-outcome, and corresponding 95% CIs (confidence intervals) and/or *p*-value, (7) papers published in English language, and (8) peer-reviewed studies. Studies reporting exclusively on brain metastases treatment were excluded. Meetings, proceedings, case reports and letters to Editors were excluded. There was no minimum sample size. When results from the same clinical trial were published several times over the selected time range, the article showing the most mature results on the primary end-point was chosen. Studies were initially screened by title and abstract; subsequently, full-text articles were analysed to verify their eligibility.

### 2.2. Study Check and Quality Rating

At the completion of article collection, 32 studies were randomly selected to perform a double review; only 6 of 1322 variables (0.4%) were found to be discordant with the original reporting (Supplementary File S1). An overall quality score (OQS) was applied to rate selected studies using a 27-item OQS from the CONSORT guidelines [15]. The rating of methodologies and results was managed by four authors (A.O., M.C., M.S., and G.N.). Data were independently rated by two authors (A.O. and M.C.) who were blinded to each other’s results (M.S. and G.N.). All discordances evidenced after revisions were resolved in a plenary discussion. Considering the predominant descriptive aim of this review, no attempt was made to reveal and quantify eventual publication bias. The impact factors of the journals reporting the selected articles were found at https://jcr.clarivate.com/jcr/home (accessed on 2 February 2022).

### 2.3. Variables

Three groups of variables were collected from selected studies: identity-, method- and outcome-related. The identifier variables were first author, year of publication, and acronym of trial. The methodological variables were clinical setting, treatment arms, primary end-point, number of patients, stratification factors in relation to disease extent, description of the oligo-metastatic disease, subgroup analysis according to the number and/or volume of metastatic disease, and definition of low-burden disease. The outcome-related variables were hazard ratios (HRs) for the primary end-point and study conclusions. When the study had both PFS and OS as primary end-points, only HR for OS was reported in the descriptive tables.

### 2.4. Data Reporting and Analysis

The analysis of the selected trials was predominantly descriptive; however, associations between disease-extent-related variables (low- versus high-burden disease) among different treatment arms were evaluated in contingency tables by use of the χ^2^ test (*p* < 0.05 were considered statistically significant). All statistical analyses were performed using MedCalc^®^ 9.3.7.0 and Excel software. According to our internal policies, the institutional review board approval and PROSPERO registration were not required for the systematic literature review.

## 3. Results

### 3.1. Study Selection

**Figure 1:**
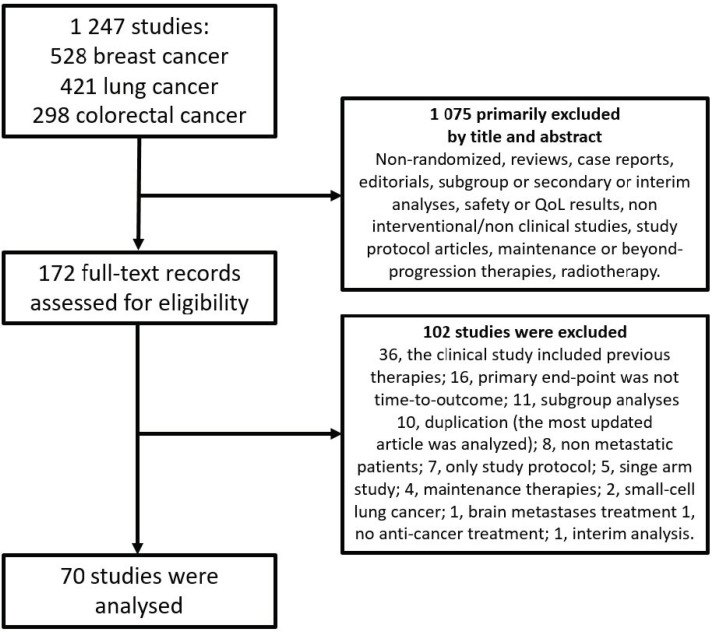
Studies selection flow-chart.

Seventy clinical studies were selected and analysed. The highest number of trials was published in 2020 (*n* = 17, 24.3%). The most frequent reasons for study exclusion, after full-text study assessment, were that the study concerned a treatment beyond first-line (*n* = 36), the non-time-to-event nature of the efficacy end-points (*n* = 16), and the non-interventional nature of the study (only secondary sub-group analyses, *n* = 11). The study selection flow-chart is reported in Figure 1. A complete list of analysed full-text articles can be viewed in Supplementary File S2.

### 3.2. Study Characteristics

The median impact factor for all studies was 28.8 (95% CI: 16.7–35.8). The mean OQS was 18.7 (95% CI: 16.9–20.8). The characteristics of the included studies are detailed in Supplementary File S3 and summarised in Table 1. Most studies dealt with non-small-cell lung (NSCLC) (*n* = 43; 61.4%) followed by breast (*n* = 17, 24.3%), and colorectal (*n* = 10; 14.3%) cancer. The median number of patients was 442 (range: 32–1486). A large portion of the studies (*n* = 29, 41.4%) focused on the effects of biologic agents (monoclonal antibodies, small molecules, and antibody-drug conjugates). PFS was the primary end-point in more than half of the studies (*n* = 44, 62.8%). Forty-five studies (64.3%) reported positive results intended as a statistically significant difference in primary end-point in favour of the experimental arm. Most studies (*n* = 48; 68.6%) did not include a placebo in the control arm.

### 3.3. Reporting of Disease Extent in the Selected Trials

The modalities of reporting the initial tumour burden in clinical trials are detailed in Supplementary File S4 and summarised in Table 2. A definition of low-burden disease was reported in 20 trials (28.6%). Disease extent was a stratification factor for randomisation in only 18 studies (25.7%). The number of patients with low-burden disease per arm was shown in 19 studies (27.1%). In all studies, no information at all was included about either the eventual enrolment of oligo-metastatic patients or the definition of oligometastatic disease. A subgroup analysis exploring outcome differences between arms according to low-burden disease and/or the number of metastatic sites was reported in 13 studies (18.6% of articles). In two studies, a significant imbalance between treatment arms in terms of the distribution of low-burden disease patients was reported (study acronyms: TURANDOT and Impassion131).

## 4. Discussion

We performed a systematic review of phase III, randomised, clinical trials in NSCLC, breast, and colorectal cancers to investigate the modalities of reporting and analysing the initial tumour volume of enrolled patients (including the eventual oligo-metastatic status).

Interestingly, no explicit and well-defined identification of oligo-metastatic disease was found in the analysed studies. The presence of a “low-burden disease” was reported in 28.6% of trials: in almost all cases, it was based on the enumeration of the number of involved organs without any additional insight into the extension of the disease (number and size of the metastases). The latter cannot be considered a surrogate of an oligo-metastatic status since a patient with only one involved organ can have both a number and total size of metastatic lesions higher than another with two or more involved sites.

Moreover, only in a low number of clinical trials was the extent of the disease a stratification factor, and in none of the examined studies was an explicit definition of oligo-metastatic disease used as exclusion criteria. In some studies, particularly in those concerning lung cancer, the stage (III vs. IV) was used as a stratification factor. However, a patient with stage IV (oligo-)metastatic disease may have less tumour burden than another with very extensive (stage III) loco-regional lymph-nodal disease. Furthermore, in 18.6% of studies, sub-group analyses did not analyse the oligo- vs. poly-metastatic status, and only the disease burden (high vs. low) was heterogeneously defined. Ultimately, if the size of either low-burden disease or oligo-metastatic patients in different study arms is different, unexpected and uncontrollable biases can affect the study results. In two studies, we reported a significant imbalance between arms in patients with low-burden disease, leading to a high probability of definitive biased results.

Could the unbalanced enrolment of genuine oligo-metastatic patients influence the prognosis of patients and consequently the results of trials? This is a crucial methodologic question that remains open. In fact, even if a certain grade of heterogeneity in terms of tumour burden among enrolled patients is perceived, the direct prognostic effect of an unbalanced distribution of oligo-metastatic patients between different treatment arms cannot be measured in our study. This is related to the complete absence of this issue in the analysed trials. In light of this, since our oncological structure (Department of Abdominal Oncology, Sub-Structure of Innovative Therapies for Abdominal Cancers) is focused on the treatment of mCRC, to provide intuitive and solid evidence on the prognostic interference of omCRC, we collected information on the treatment and outcomes of 112 consecutive mCRC with clinical characteristics permissive for inclusion in phase III studies. A strict time range (the last three years) was applied to reduce therapeutic and methodological heterogeneity. A detailed description of this cohort is beyond the scope of this article. However, there was an evident and statistically significant difference between pmCRC and omCRC patients in terms of survival (Supplementary File S5). Some patients were indeed enrolled in clinical trials. Interestingly, the prognosis of this cohort was positively influenced by omCRC patients. In particular, the median survival of the “entire”/”undifferentiated” mCRC cohort (including also omCRC patients) was 24.0 months compared to 22.0 months for the true pmCRC patients (excluding omCRC patients). Therefore, the contamination of omCRC patients improved the prognosis (+2.0 months). This can be particularly relevant from a statistical point of view in large clinical trials where a 2-month gain is sufficient to induce misleading positive results [16].

Moreover, it has been demonstrated that immunotherapies are more active in patients with low-burden diseases due to the higher number of immunosuppressing cells in larger masses [17]. In this case, the absence of oligo-metastatic or low-burden disease identification can interfere with the efficacy assessment of the different drugs in the different arms.

It is increasingly evident that the initial tumour burden of metastatic patients is a crucial prognostic factor and that oligo-metastatic disease represents a specific clinical entity with slower progression and increased sensitivity to treatments. Therefore, the clinical impact of these patients’ fractions could be relevant from both prognostic/therapeutic and methodologic perspectives. In our opinion, the burden of disease [4,6,9] remains the most solid and simple way to identify the potential oligo-metastatic status, as specific biological and molecular characteristics are still elusive and unknown.

## 5. Conclusions

We suggest that a better evaluation of tumour burden in future clinical trials (including the assessment of oligo-metastatic disease and complete estimates of tumour volume) could increase the soundness of phase III study results, particularly in those based on the use of biological drugs. In this context, in addition to basal clinical and radiological evaluations, advanced computational tools for the automated quantification of tumour volume could be integrated in clinical trial designs, being useful for both patient stratification at enrolment and the better interpretation of final efficacy data. Moreover, the harmonisation of the definition and reporting of oligo-metastatic and low-burden diseases is strongly required through consensus meetings.

## Figures and Tables

**Table 1:** Characteristics of the selected phase III clinical trials (a total of 70 studies were analysed).

Characteristic	No.	%
Type of cancer		
Non-small-cell lung cancer	43	61.4
Breast	17	24.3
Colorectal	10	14.3
Sample size		
Median	442	
Range	32–1486	
Type of therapy		
Signal inhibitors (including antibodies and small molecules)	17	24.3
Immuno-therapy	12	17.1
Chemotherapy	10	14.3
Associations	31	44.3
Chemotherapy + signal inhibitors	16	
Chemotherapy + immuno-therapy	8	
Signal inhibitors + hormone-therapy	4	
Chemotherapy + signal inhibitors + immuno-therapy	3	
Primary end-points		
PFS	44	62.8
OS	13	18.6
PFS and OS	13	18.6
Study conclusions		
Positive	45	64.3
Negative	17	24.3
Non-inferior	7	10.0
Equivalent	1	1.4
Inclusion of placebo in the control arm		
Yes	22	31.4
No	48	68.6

**Table 2:** Modalities of reporting tumour burden.

Characteristics	No.	%
Reporting of low-burden disease		
Yes	20	28.6
No	50	71.4
Identification modality		
No. of metastatic sites	19	27.1
Tumour diameter	1	1.4
Not reported	50	71.4
Disease extent as a stratification factor		
Yes	18	25.7
No	52	74.3
Low-burden disease *per* treatment arm		
Yes	19	27.1
No	51	72.9
Reporting of oligo-metastatic disease		
Yes	0	0
No	70	100
Subgroup analysis according to disease extent		
Yes	13	18.6
No	57	81.3

## Data Availability

The data presented in this study are available in this article.

## Supplementary Materials

The following supporting information can be downloaded at: https://www.mdpi.com/article/10.3390/cancers14133262/s1, File S1: Discordances between the original data and those inserted in the database, File S2: Complete list of articles, File S3: Description of study identifiers, treatment arms, and outcome-related variables, File S4: Description of disease-extent-related variables in the selected studies, Supplementary File S5: Effect of oligo-metastatic colorectal cancer patients on the prognosis of a cohort with clinical characteristics permissive for inclusion in phase III trials.
