# Supplementary material for: Tumour Burden Reporting in Phase III Clinical Trials of Metastatic Lung, Breast, and Colorectal Cancers: A Systematic Review
Source: Cancers (Basel). 2022 Jul 3;14(13):3262. doi: 10.3390/cancers14133262 (PMC9264965; doi:10.3390/cancers14133262)
Supplement: Supplementary file 1 [file cancers-14-03262-s001.zip › Supplementary File 1.pdf]

Supplementary file 1. Discordances between the original data and those inserted in the database (in 32 studies as revealed after double-checking by authors AO and MC).

Study (acronym)	Disease	Original data (as reported in the article)	Data inserted in the database	Author	Type of discordance/ error	Action
J-ALEX	NSCLC	Arm 1: Alectinib	Aletinib	MS	Typo	Only correction
UMIN000011460	NSCLC	Study conclusion: Equivalent	Positive	MS	Interpretation	Revision of all "study conclusion" interpretations for all 70 studies
CameL	NSCLC	Year of publication: 2020	2021	MS	Typo	Only correction
EMPOWER-Lung 1	NSCLC	*P* for OS: 0.0002	0.002	VDL	Typo	Only correction
SELECT-BC	Breast	HR for PFS, 95% CI inferior limit: 0.86	0.76	FP	Typo	Only correction
IMpassion131	Breast	Low burden disease: <4 metastatic sites	No	MS	Interpretation	Revision of all "low-burden disease" interpretations for all 70 studies
