# Supplementary material for: Tumour Burden Reporting in Phase III Clinical Trials of Metastatic Lung, Breast, and Colorectal Cancers: A Systematic Review
Source: Cancers (Basel). 2022 Jul 3;14(13):3262. doi: 10.3390/cancers14133262 (PMC9264965; doi:10.3390/cancers14133262)
Supplement: Supplementary file 1 [file cancers-14-03262-s001.zip › Supplementary File 2.pdf]

Supplementary file 2: Complete list of full-text articles analysed

LUNG CANCER

Rodríguez-Abreu D, Powell SF, Hochmair MJ, Gadgeel S, Esteban E, Felip E, Speranza G, De Angelis F, Dómine M, Cheng SY, Bischoff HG, Peled N, Reck M, Hui R, Garon EB, Boyer M, Kurata T, Yang J, Pietanza MC, Souza F, Garassino MC. Pemetrexed plus platinum with or without pembrolizumab in patients with previously untreated metastatic nonsquamous NSCLC: protocol-specified final analysis from KEYNOTE-189. Ann Oncol. 2021 Jul;32(7):881-895. doi: 10.1016/j.annonc.2021.04.008. Epub 2021 Apr 22. PMID: 33894335.

Lu S, Chen G, Sun Y, Sun S, Chang J, Yao Y, Chen Z, Ye F, Lu J, Shi J, He J, Liu X, Zhang Y, Liu Z, Fang J, Cheng Y, Hu C, Mao W, Hu Y, Gong Y, Shan L, Yang Z, Song Y, Li W, Bai C, Wang B, Ma R, Zheng Z, Liu M, Jie Z, Cao L, Liao W, Pan H, Huang D, Chen Y, Yang J, Qin S, Ma S, Liang L, Liu Z, Zhou J, Tao M, Huang Y, Qiu F, Huang Y, Guan S, Peng M, Su W. A Phase III, randomized, double-blind, placebo-controlled, multicenter study of fruquintinib in Chinese patients with advanced nonsquamous non-small-cell lung cancer - The FALUCA study. Lung Cancer. 2020 Aug;146:252-262. doi: 10.1016/j.lungcan.2020.06.016. Epub 2020 Jun 20. PMID: 32592986.

Seto T, Azuma K, Yamanaka T, Sugawara S, Yoshioka H, Wakuda K, Atagi S, Iwamoto Y, Hayashi H, Okamoto I, Saka H, Mitsuoka S, Fujimoto D, Nishino K, Horiike A, Daga H, Sone T, Yamamoto N, Nakagawa K, Nakanishi Y. Randomized Phase III Study of Continuation Maintenance Bevacizumab With or Without Pemetrexed in Advanced Nonsquamous Non-Small-Cell Lung Cancer: COMPASS (WJOG5610L). J Clin Oncol. 2020 Mar 10;38(8):793-803. doi: 10.1200/JCO.19.01494. Epub 2019 Dec 27. PMID: 31880966.

Zhou C, Kim SW, Reungwetwattana T, Zhou J, Zhang Y, He J, Yang JJ, Cheng Y, Lee SH, Bu L, Xu T, Yang L, Wang C, Liu T, Morcos PN, Lu Y, Zhang L. Alectinib versus crizotinib in untreated Asian patients with anaplastic lymphoma kinase-positive non-small-cell lung cancer (ALESIA): a randomised phase 3 study. Lancet Respir Med. 2019 May;7(5):437-446. doi: 10.1016/S2213-2600(19)30053-0. Epub 2019 Apr 10. PMID: 30981696.

Wu YL, Lu S, Lu Y, Zhou J, Shi YK, Sriuranpong V, Ho JCM, Ong CK, Tsai CM, Chung CH, Wilner KD, Tang Y, Masters ET, Selaru P, Mok TS. Results of PROFILE 1029, a Phase III Comparison of First-Line Crizotinib versus Chemotherapy in East Asian Patients with ALK-Positive Advanced Non-Small Cell Lung Cancer. J Thorac Oncol. 2018 Oct;13(10):1539-1548. doi: 10.1016/j.jtho.2018.06.012. Epub 2018 Aug 14. PMID: 29966800.

Solomon BJ, Kim DW, Wu YL, Nakagawa K, Mekhail T, Felip E, Cappuzzo F, Paolini J, Usari T, Tang Y, Wilner KD, Blackhall F, Mok TS. Final Overall Survival Analysis From a Study Comparing First-Line Crizotinib Versus Chemotherapy in ALK-Mutation-Positive Non-Small-Cell Lung Cancer. J Clin Oncol. 2018 Aug 1;36(22):2251-2258. doi: 10.1200/JCO.2017.77.4794. Epub 2018 May 16. PMID: 29768118.

Hanna NH, Kaiser R, Sullivan RN, Aren OR, Ahn MJ, Tiangco B, Voccia I, Pawel JV, Kovcin V, Agulnik J, Gaschler-Markefski B, Barrueco J, Sikken P, Schloss C, Kim JH; LUME-Lung 2 Study group. Nintedanib plus pemetrexed versus placebo plus pemetrexed in patients with relapsed or refractory, advanced non-small cell lung cancer (LUME-Lung 2): A randomized, double-blind, phase III trial. Lung Cancer. 2016 Dec;102:65-73. doi: 10.1016/j.lungcan.2016.10.011. Epub 2016 Oct 27. PMID: 27987591.

Ramalingam SS, Novello S, Guclu SZ, Bentsion D, Zvirbule Z, Szilasi M, Bernabe R, Syrigos K, Byers LA, Clingan P, Bar J, Vokes EE, Govindan R, Dunbar M, Ansell P, He L, Huang X, Sehgal V, Glasgow J, Bach BA, Mazieres J. Veliparib in Combination With Platinum-Based Chemotherapy for First-Line Treatment of Advanced Squamous Cell Lung Cancer: A Randomized, Multicenter Phase III Study. J Clin Oncol. 2021 Nov 10;39(32):3633-3644. doi: 10.1200/JCO.20.03318. Epub 2021 Aug 26. PMID: 34436928; PMCID: PMC8577684.

Steendam CMJ, Peric R, van Walree NC, Youssef M, Schramel FMNH, Brocken P, van Putten JWG, van der Noort V, Veerman GDM, Koolen SLW, Groen HJM, Dingemans AC, Mathijssen RHJ, Smit EF, Aerts JGJV; NVALT Study Group. Randomized phase III study of docetaxel versus docetaxel plus intercalated erlotinib in patients with relapsed non-squamous non-small cell lung carcinoma. Lung Cancer. 2021 Oct;160:44-49. doi: 10.1016/j.lungcan.2021.08.002. Epub 2021 Aug 4. PMID: 34403911.

Zhou C, Wu L, Fan Y, Wang Z, Liu L, Chen G, Zhang L, Huang D, Cang S, Yang Z, Zhou J, Zhou C, Li B, Li J, Fan M, Cui J, Li Y, Zhao H, Fang J, Xue J, Hu C, Sun P, Du Y, Zhou H, Wang S, Zhang W. Sintilimab Plus Platinum and Gemcitabine as First-Line Treatment for Advanced or Metastatic Squamous NSCLC: Results From a Randomized, Double-Blind, Phase 3 Trial (ORIENT-12). J Thorac Oncol. 2021 Sep;16(9):1501-1511. doi: 10.1016/j.jtho.2021.04.011. Epub 2021 May 25. PMID: 34048947.

Lu S, Wang J, Yu Y, Yu X, Hu Y, Ai X, Ma Z, Li X, Zhuang W, Liu Y, Li W, Cui J, Wang D, Liao W, Zhou J, Wang Z, Sun Y, Qiu X, Gao J, Bao Y, Liang L, Wang M. Tislelizumab Plus Chemotherapy as First-Line Treatment for Locally Advanced or Metastatic Nonsquamous NSCLC (RATIONALE 304): A Randomized Phase 3 Trial. J Thorac Oncol. 2021 Sep;16(9):1512-1522. doi: 10.1016/j.jtho.2021.05.005. Epub 2021 May 23. PMID: 34033975.

Wang J, Lu S, Yu X, Hu Y, Sun Y, Wang Z, Zhao J, Yu Y, Hu C, Yang K, Feng G, Ying K, Zhuang W, Zhou J, Wu J, Leaw SJ, Zhang J, Lin X, Liang L, Yang N. Tislelizumab Plus Chemotherapy vs Chemotherapy Alone as First-line Treatment for Advanced Squamous Non-Small-Cell Lung Cancer: A Phase 3 Randomized Clinical Trial. JAMA Oncol. 2021 May 1;7(5):709-717. doi: 10.1001/jamaoncol.2021.0366. PMID: 33792623; PMCID: PMC8017481.

Sezer A, Kilickap S, Gümüş M, Bondarenko I, Özgüroğlu M, Gogishvili M, Turk HM, Cicin I, Bentsion D, Gladkov O, Clingan P, Sriuranpong V, Rizvi N, Gao B, Li S, Lee S, McGuire K, Chen CI, Makharadze T, Paydas S, Nechaeva M, Seebach F, Weinreich DM, Yancopoulos GD, Gullo G, Lowy I, Rietschel P. Cemiplimab monotherapy for first-line treatment of advanced non-small-cell lung cancer with PD-L1 of at least 50%: a multicentre, open-label, global, phase 3, randomised, controlled trial. Lancet. 2021 Feb 13;397(10274):592-604. doi: 10.1016/S0140-6736(21)00228-2. PMID: 33581821.

Cheng Y, He Y, Li W, Zhang HL, Zhou Q, Wang B, Liu C, Walding A, Saggese M, Huang X, Fan M, Wang J, Ramalingam SS. Osimertinib Versus Comparator EGFR TKI as First-Line Treatment for EGFR-Mutated Advanced NSCLC: FLAURA China, A Randomized Study. Target Oncol. 2021 Mar;16(2):165-176. doi: 10.1007/s11523-021-00794-6. Epub 2021 Feb 5. PMID: 33544337; PMCID: PMC7935816.

Paz-Ares L, Ciuleanu TE, Cobo M, Schenker M, Zurawski B, Menezes J, Richardet E, Bennouna J, Felip E, Juan-Vidal O, Alexandru A, Sakai H, Lingua A, Salman P, Souquet PJ, De Marchi P, Martin C, Pérol M, Scherpereel A, Lu S, John T, Carbone DP, Meadows-Shropshire S, Agrawal S, Oukessou A, Yan J, Reck M. First-line nivolumab plus ipilimumab combined with two cycles of chemotherapy in patients with non-small-cell lung cancer (CheckMate 9LA): an international, randomised, open-label, phase 3 trial. Lancet Oncol. 2021 Feb;22(2):198-211. doi: 10.1016/S1470-2045(20)30641-0. Epub 2021 Jan 18. Erratum in: Lancet Oncol. 2021 Mar;22(3):e92. PMID: 33476593.

Liu SV, Reck M, Mansfield AS, Mok T, Scherpereel A, Reinmuth N, Garassino MC, De Castro Carpeno J, Califano R, Nishio M, Orlandi F, Alatorre-Alexander J, Leal T, Cheng Y, Lee JS, Lam S, McCleland M, Deng Y, Phan S, Horn L. Updated Overall Survival and PD-L1 Subgroup Analysis of Patients With Extensive-Stage Small-Cell Lung Cancer Treated With Atezolizumab, Carboplatin, and Etoposide (IMpower133). J Clin Oncol. 2021 Feb 20;39(6):619-630. doi: 10.1200/JCO.20.01055. Epub 2021 Jan 13. PMID: 33439693; PMCID: PMC8078320.

Zhou C, Chen G, Huang Y, Zhou J, Lin L, Feng J, Wang Z, Shu Y, Shi J, Hu Y, Wang Q, Cheng Y, Wu F, Chen J, Lin X, Wang Y, Huang J, Cui J, Cao L, Liu Y,

Zhang Y, Pan Y, Zhao J, Wang L, Chang J, Chen Q, Ren X, Zhang W, Fan Y, He Z, Fang J, Gu K, Dong X, Zhang T, Shi W, Zou J; CameL Study Group. Camrelizumab plus carboplatin and pemetrexed versus chemotherapy alone in chemotherapy-naive patients with advanced non-squamous non-small-cell lung cancer (CameL): a randomised, open-label, multicentre, phase 3 trial. Lancet Respir Med. 2021 Mar;9(3):305-314. doi: 10.1016/S2213-2600(20)30365-9. Epub 2020 Dec 18. PMID: 33347829.

Nishio M, Barlesi F, West H, Ball S, Bordoni R, Cobo M, Longeras PD, Goldschmidt J Jr, Novello S, Orlandi F, Sanborn RE, Szalai Z, Ursol G, Mendus D, Wang L, Wen X, McCleland M, Hoang T, Phan S, Socinski MA. Atezolizumab Plus Chemotherapy for First-Line Treatment of Nonsquamous NSCLC: Results From the Randomized Phase 3 IMpower132 Trial. J Thorac Oncol. 2021 Apr;16(4):653-664. doi: 10.1016/j.jtho.2020.11.025. Epub 2020 Dec 14. PMID: 33333328.

Mok TS, Cheng Y, Zhou X, Lee KH, Nakagawa K, Niho S, Chawla A, Rosell R, Corral J, Migliorino MR, Pluzanski A, Noonan K, Tang Y, Pastel M, Wilner KD, Wu YL. Updated Overall Survival in a Randomized Study Comparing Dacomitinib with Gefitinib as First-Line Treatment in Patients with Advanced Non-Small-Cell Lung Cancer and EGFR-Activating Mutations. Drugs. 2021 Feb;81(2):257-266. doi: 10.1007/s40265-020-01441-6. PMID: 33331989; PMCID: PMC7932969.

Shaw AT, Bauer TM, de Marinis F, Felip E, Goto Y, Liu G, Mazieres J, Kim DW, Mok T, Polli A, Thurm H, Calella AM, Peltz G, Solomon BJ; CROWN Trial Investigators. First-Line Lorlatinib or Crizotinib in Advanced <i>ALK</i>-Positive Lung Cancer. N Engl J Med. 2020 Nov 19;383(21):2018-2029. doi: 10.1056/NEJMoa2027187. PMID: 33207094.

Shi M, Gu A, Tu H, Huang C, Wang H, Yu Z, Wang X, Cao L, Shu Y, Wang H, Yang R, Li X, Chang J, Hu Y, Shen P, Hu Y, Guo Z, Tao M, Zhang Y, Liu X, Sun Q, Zhang X, Jiang Z, Zhao J, Chen F, Yu H, Zhang W, Sun J, Li D, Zhou J, Han B, Wu YL. Comparing nanoparticle polymeric micellar paclitaxel and solvent-based paclitaxel as first-line treatment of advanced non-small-cell lung cancer: an open-label, randomized, multicenter, phase III trial. Ann Oncol. 2021 Jan;32(1):85-96. doi: 10.1016/j.annonc.2020.10.479. Epub 2020 Oct 29. PMID: 33130217.

Spigel DR, Jotte RM, Aix SP, Gressot L, Morgensztern D, McCleod M, Socinski MA, Daniel D, Juan-Vidal O, Mileham KF, West H, Page R, Reinmuth N, Knoble J, Chen T, Bhore R, Wolfsteiner M, Ong TJ, Gridelli C, Thomas M; ABOUND.sqm Investigators. Nanoparticle Albumin-bound Paclitaxel Plus Carboplatin Induction Followed by Nanoparticle Albumin-bound Paclitaxel Maintenance in Squamous Non-Small-cell Lung Cancer (ABOUND.sqm): A Phase III Randomized Clinical Trial. Clin

Lung Cancer. 2021 Jan;22(1):6-15.e4. doi: 10.1016/j.cllc.2020.09.007. Epub 2020 Sep 18. PMID: 33097414.

Herbst RS, Giaccone G, de Marinis F, Reinmuth N, Vergnenegre A, Barrios CH, Morise M, Felip E, Andric Z, Geater S, Özgüroğlu M, Zou W, Sandler A, Enquist I, Komatsubara K, Deng Y, Kuriki H, Wen X, McCleland M, Mocci S, Jassem J, Spigel DR. Atezolizumab for First-Line Treatment of PD-L1-Selected Patients with NSCLC. N Engl J Med. 2020 Oct 1;383(14):1328-1339. doi: 10.1056/NEJMoa1917346. PMID: 32997907.

Wu YL, Tsuboi M, He J, John T, Grohe C, Majem M, Goldman JW, Laktionov K, Kim SW, Kato T, Vu HV, Lu S, Lee KY, Akewanlop C, Yu CJ, de Marinis F, Bonanno L, Domine M, Shepherd FA, Zeng L, Hodge R, Atasoy A, Rukazenkov Y, Herbst RS; ADAURA Investigators. Osimertinib in Resected <i>EGFR</i>-Mutated Non-Small-Cell Lung Cancer. N Engl J Med. 2020 Oct 29;383(18):1711-1723. doi: 10.1056/NEJMoa2027071. Epub 2020 Sep 19. PMID: 32955177.

Yang Y, Wang Z, Fang J, Yu Q, Han B, Cang S, Chen G, Mei X, Yang Z, Ma R, Bi M, Ren X, Zhou J, Li B, Song Y, Feng J, Li J, He Z, Zhou R, Li W, Lu Y, Wang Y, Wang L, Yang N, Zhang Y, Yu Z, Zhao Y, Xie C, Cheng Y, Zhou H, Wang S, Zhu D, Zhang W, Zhang L. Efficacy and Safety of Sintilimab Plus Pemetrexed and Platinum as First-Line Treatment for Locally Advanced or Metastatic Nonsquamous NSCLC: a Randomized, Double-Blind, Phase 3 Study (Oncology pRogram by InnovENT anti-PD-1-11). J Thorac Oncol. 2020 Oct;15(10):1636-1646. doi: 10.1016/j.jtho.2020.07.014. Epub 2020 Aug 8. PMID: 32781263.

Camidge DR, Kim HR, Ahn MJ, Yang JCH, Han JY, Hochmair MJ, Lee KH, Delmonte A, García Campelo MR, Kim DW, Griesinger F, Felip E, Califano R, Spira A, Gettinger SN, Tiseo M, Lin HM, Gupta N, Hanley MJ, Ni Q, Zhang P, Popat S. Brigatinib Versus Crizotinib in Advanced ALK Inhibitor-Naive ALK-Positive Non-Small Cell Lung Cancer: Second Interim Analysis of the Phase III ALTA-1L Trial. J Clin Oncol. 2020 Nov 1;38(31):3592-3603. doi: 10.1200/JCO.20.00505. Epub 2020 Aug 11. PMID: 32780660; PMCID: PMC7605398.

Peters S, Danson S, Hasan B, Dafni U, Reinmuth N, Majem M, Tournoy KG, Mark MT, Pless M, Cobo M, Rodriguez-Abreu D, Falchero L, Moran T, Ortega Granados AL, Monnet I, Mohorcic K, Sureda BM, Betticher D, Demedts I, Macias JA, Cuffe S, Luciani A, Sanchez JG, Curioni-Fontecedro A, Gautschi O, Price G, Coate L, von Moos R, Zielinski C, Provencio M, Menis J, Ruepp B, Pochesci A, Roschitzki-Voser H, Besse B, Rabaglio M, O'Brien MER, Stahel RA. A Randomized Open-Label Phase III Trial Evaluating the Addition of Denosumab to Standard First-Line Treatment in Advanced NSCLC: The European Thoracic Oncology Platform (ETOP) and European Organisation for Research and Treatment of Cancer (EORTC) SPLENDOUR Trial. J

Thorac Oncol. 2020 Oct;15(10):1647-1656. doi: 10.1016/j.jtho.2020.06.011. Epub 2020 Jun 18. PMID: 32565388.

Jotte R, Cappuzzo F, Vynnychenko I, Stroyakovskiy D, Rodríguez-Abreu D, Hussein M, Soo R, Conter HJ, Kozuki T, Huang KC, Graupner V, Sun SW, Hoang T, Jessop H, McCleland M, Ballinger M, Sandler A, Socinski MA. Atezolizumab in Combination With Carboplatin and Nab-Paclitaxel in Advanced Squamous NSCLC (IMpower131): Results From a Randomized Phase III Trial. J Thorac Oncol. 2020 Aug;15(8):1351-1360. doi: 10.1016/j.jtho.2020.03.028. Epub 2020 Apr 14. PMID: 32302702.

Rizvi NA, Cho BC, Reinmuth N, Lee KH, Luft A, Ahn MJ, van den Heuvel MM, Cobo M, Vicente D, Smolin A, Moiseyenko V, Antonia SJ, Le Moulec S, Robinet G, Natale R, Schneider J, Shepherd FA, Geater SL, Garon EB, Kim ES, Goldberg SB, Nakagawa K, Raja R, Higgs BW, Boothman AM, Zhao L, Scheuring U, Stockman PK, Chand VK, Peters S; MYSTIC Investigators. Durvalumab With or Without Tremelimumab vs Standard Chemotherapy in First-line Treatment of Metastatic Non-Small Cell Lung Cancer: The MYSTIC Phase 3 Randomized Clinical Trial. JAMA Oncol. 2020 May 1;6(5):661-674. doi: 10.1001/jamaoncol.2020.0237. Erratum in: JAMA Oncol. 2020 Nov 1;6(11):1815. PMID: 32271377; PMCID: PMC7146551.

Okamoto I, Nokihara H, Nomura S, Niho S, Sugawara S, Horinouchi H, Azuma K, Yoneshima Y, Murakami H, Hosomi Y, Atagi S, Ozaki T, Horiike A, Fujita Y, Okamoto H, Ando M, Yamamoto N, Ohe Y, Nakagawa K. Comparison of Carboplatin Plus Pemetrexed Followed by Maintenance Pemetrexed With Docetaxel Monotherapy in Elderly Patients With Advanced Nonsquamous Non-Small Cell Lung Cancer: A Phase 3 Randomized Clinical Trial. JAMA Oncol. 2020 May 1;6(5):e196828. doi: 10.1001/jamaoncol.2019.6828. Epub 2020 May 14. PMID: 32163097; PMCID: PMC7068674.

Garassino MC, Gadgeel S, Esteban E, Felip E, Speranza G, Domine M, Hochmair MJ, Powell S, Cheng SY, Bischoff HG, Peled N, Reck M, Hui R, Garon EB, Boyer M, Wei Z, Burke T, Pietanza MC, Rodríguez-Abreu D. Patient-reported outcomes following pembrolizumab or placebo plus pemetrexed and platinum in patients with previously untreated, metastatic, non-squamous non-small-cell lung cancer (KEYNOTE-189): a multicentre, double-blind, randomised, placebo-controlled, phase 3 trial. Lancet Oncol. 2020 Mar;21(3):387-397. doi: 10.1016/S1470-2045(19)30801-0. Epub 2020 Feb 6. PMID: 32035514.

Pillai RN, Fennell DA, Kovcin V, Ciuleanu TE, Ramlau R, Kowalski D, Schenker M, Yalcin I, Teofilovici F, Vukovic VM, Ramalingam SS. Randomized Phase III Study of Ganetespib, a Heat Shock Protein 90 Inhibitor, With Docetaxel Versus Docetaxel in Advanced Non-Small-Cell Lung Cancer (GALAXY-2). J Clin Oncol. 2020

Feb 20;38(6):613-622. doi: 10.1200/JCO.19.00816. Epub 2019 Dec 12. PMID: 31829907.

Ramalingam SS, Vansteenkiste J, Planchard D, Cho BC, Gray JE, Ohe Y, Zhou C, Reungwetwattana T, Cheng Y, Chewaskulyong B, Shah R, Cobo M, Lee KH, Cheema P, Tiseo M, John T, Lin MC, Imamura F, Kurata T, Todd A, Hodge R, Saggese M, Rukazenkov Y, Soria JC; FLAURA Investigators. Overall Survival with Osimertinib in Untreated, <i>EGFR</i>-Mutated Advanced NSCLC. N Engl J Med. 2020 Jan 2;382(1):41-50. doi: 10.1056/NEJMoa1913662. Epub 2019 Nov 21. PMID: 31751012.

Hosomi Y, Morita S, Sugawara S, Kato T, Fukuhara T, Gemma A, Takahashi K, Fujita Y, Harada T, Minato K, Takamura K, Hagiwara K, Kobayashi K, Nukiwa T, Inoue A; North-East Japan Study Group. Gefitinib Alone Versus Gefitinib Plus Chemotherapy for Non-Small-Cell Lung Cancer With Mutated Epidermal Growth Factor Receptor: NEJ009 Study. J Clin Oncol. 2020 Jan 10;38(2):115-123. doi: 10.1200/JCO.19.01488. Epub 2019 Nov 4. PMID: 31682542.

Hellmann MD, Paz-Ares L, Bernabe Caro R, Zurawski B, Kim SW, Carcereny Costa E, Park K, Alexandru A, Lupinacci L, de la Mora Jimenez E, Sakai H, Albert I, Vergnenegre A, Peters S, Syrigos K, Barlesi F, Reck M, Borghaei H, Brahmer JR, O'Byrne KJ, Geese WJ, Bhagavatheeswaran P, Rabindran SK, Kasinathan RS, Nathan FE, Ramalingam SS. Nivolumab plus Ipilimumab in Advanced Non-Small-Cell Lung Cancer. N Engl J Med. 2019 Nov 21;381(21):2020-2031. doi: 10.1056/NEJMoa1910231. Epub 2019 Sep 28. PMID: 31562796.

Yoshioka H, Shimokawa M, Seto T, Morita S, Yatabe Y, Okamoto I, Tsurutani J, Satouchi M, Hirashima T, Atagi S, Shibata K, Saito H, Toyooka S, Yamamoto N, Nakagawa K, Mitsudomi T. Final overall survival results of WJTOG3405, a randomized phase III trial comparing gefitinib versus cisplatin with docetaxel as the first-line treatment for patients with stage IIIB/IV or postoperative recurrent EGFR mutation-positive non-small-cell lung cancer. Ann Oncol. 2019 Dec 1;30(12):1978-1984. doi: 10.1093/annonc/mdz399. PMID: 31553438.

Noronha V, Patil VM, Joshi A, Menon N, Chougule A, Mahajan A, Janu A, Purandare N, Kumar R, More S, Goud S, Kadam N, Daware N, Bhattacharjee A, Shah S, Yadav A, Trivedi V, Behel V, Dutt A, Banavali SD, Prabhash K. Gefitinib Versus Gefitinib Plus Pemetrexed and Carboplatin Chemotherapy in <i>EGFR</i>-Mutated Lung Cancer. J Clin Oncol. 2020 Jan 10;38(2):124-136. doi: 10.1200/JCO.19.01154. Epub 2019 Aug 14. PMID: 31411950.

Reck M, Schenker M, Lee KH, Provencio M, Nishio M, Lesniewski-Kmak K, Sangha R, Ahmed S, Raimbourg J, Feeney K, Corre R, Franke FA, Richardet E, Penrod JR, Yuan Y, Nathan FE, Bhagavatheeswaran P, DeRosa M, Taylor F, Lawrance R, Brahmer

J. Nivolumab plus ipilimumab versus chemotherapy as first-line treatment in advanced non-small-cell lung cancer with high tumour mutational burden: patient-reported outcomes results from the randomised, open-label, phase III CheckMate 227 trial. Eur J Cancer. 2019 Jul;116:137-147. doi: 10.1016/j.ejca.2019.05.008. Epub 2019 Jun 11. PMID: 31195357.

West H, McCleod M, Hussein M, Morabito A, Rittmeyer A, Conter HJ, Kopp HG, Daniel D, McCune S, Mekhail T, Zer A, Reinmuth N, Sadiq A, Sandler A, Lin W, Ochi Lohmann T, Archer V, Wang L, Kowanetz M, Cappuzzo F. Atezolizumab in combination with carboplatin plus nab-paclitaxel chemotherapy compared with chemotherapy alone as first-line treatment for metastatic non-squamous non-small-cell lung cancer (IMpower130): a multicentre, randomised, open-label, phase 3 trial. Lancet Oncol. 2019 Jul;20(7):924-937. doi: 10.1016/S1470-2045(19)30167-6. Epub 2019 May 20. PMID: 31122901.

Kelly RJ, Shepherd FA, Krivoshik A, Jie F, Horn L. A phase III, randomized, open-label study of ASP8273 versus erlotinib or gefitinib in patients with advanced stage IIIB/IV non-small-cell lung cancer. Ann Oncol. 2019 Jul 1;30(7):1127-1133. doi: 10.1093/annonc/mdz128. PMID: 31070709; PMCID: PMC6736319.

Mok TSK, Wu YL, Kudaba I, Kowalski DM, Cho BC, Turna HZ, Castro G Jr, Srimuninnimit V, Laktionov KK, Bondarenko I, Kubota K, Lubiniecki GM, Zhang J, Kush D, Lopes G; KEYNOTE-042 Investigators. Pembrolizumab versus chemotherapy for previously untreated, PD-L1-expressing, locally advanced or metastatic non-small-cell lung cancer (KEYNOTE-042): a randomised, open-label, controlled, phase 3 trial. Lancet. 2019 May 4;393(10183):1819-1830. doi: 10.1016/S0140-6736(18)32409-7. Epub 2019 Apr 4. PMID: 30955977.

Camidge DR, Dziadziuszko R, Peters S, Mok T, Noe J, Nowicka M, Gadgeel SM, Cheema P, Pavlakis N, de Marinis F, Cho BC, Zhang L, Moro-Sibilot D, Liu T, Bordogna W, Balas B, Müller B, Shaw AT. Updated Efficacy and Safety Data and Impact of the EML4-ALK Fusion Variant on the Efficacy of Alectinib in Untreated ALK-Positive Advanced Non-Small Cell Lung Cancer in the Global Phase III ALEX Study. J Thorac Oncol. 2019 Jul;14(7):1233-1243. doi: 10.1016/j.jtho.2019.03.007. Epub 2019 Mar 20. Erratum in: J Thorac Oncol. 2019 Nov;14(11):2023. PMID: 30902613.

Reck M, Rodríguez-Abreu D, Robinson AG, Hui R, Csőszi T, Fülöp A, Gottfried M, Peled N, Tafreshi A, Cuffe S, O'Brien M, Rao S, Hotta K, Vandormael K, Riccio A, Yang J, Pietanza MC, Brahmer JR. Updated Analysis of KEYNOTE-024: Pembrolizumab Versus Platinum-Based Chemotherapy for Advanced Non-Small-Cell Lung Cancer With PD-L1 Tumor Proportion Score of 50% or Greater. J Clin Oncol.

2019 Mar 1;37(7):537-546. doi: 10.1200/JCO.18.00149. Epub 2019 Jan 8. PMID: 30620668.

von Pawel J, Bordoni R, Satouchi M, Fehrenbacher L, Cobo M, Han JY, Hida T, Moro-Sibilot D, Conkling P, Gandara DR, Rittmeyer A, Gandhi M, Yu W, Matheny C, Patel H, Sandler A, Ballinger M, Kowanetz M, Park K. Long-term survival in patients with advanced non-small-cell lung cancer treated with atezolizumab versus docetaxel: Results from the randomised phase III OAK study. Eur J Cancer. 2019 Jan;107:124-132. doi: 10.1016/j.ejca.2018.11.020. Epub 2018 Dec 17. PMID: 30562710.

Camidge DR, Kim HR, Ahn MJ, Yang JC, Han JY, Lee JS, Hochmair MJ, Li JY, Chang GC, Lee KH, Gridelli C, Delmonte A, Garcia Campelo R, Kim DW, Bearz A, Griesinger F, Morabito A, Felip E, Califano R, Ghosh S, Spira A, Gettinger SN, Tiseo M, Gupta N, Haney J, Kerstein D, Popat S. Brigatinib versus Crizotinib in ALK-Positive Non-Small-Cell Lung Cancer. N Engl J Med. 2018 Nov 22;379(21):2027-2039. doi: 10.1056/NEJMoa1810171. Epub 2018 Sep 25. PMID: 30280657.

Paz-Ares L, Luft A, Vicente D, Tafreshi A, Gümüş M, Mazières J, Hermes B, Çay Şenler F, Csőszi T, Fülöp A, Rodríguez-Cid J, Wilson J, Sugawara S, Kato T, Lee KH, Cheng Y, Novello S, Halmos B, Li X, Lubiniecki GM, Piperdi B, Kowalski DM; KEYNOTE-407 Investigators. Pembrolizumab plus Chemotherapy for Squamous Non-Small-Cell Lung Cancer. N Engl J Med. 2018 Nov 22;379(21):2040-2051. doi: 10.1056/NEJMoa1810865. Epub 2018 Sep 25. PMID: 30280635.

Gadgeel S, Peters S, Mok T, Shaw AT, Kim DW, Ou SI, Pérol M, Wrona A, Novello S, Rosell R, Zeaiter A, Liu T, Nüesch E, Balas B, Camidge DR. Alectinib versus crizotinib in treatment-naive anaplastic lymphoma kinase-positive (ALK+) non-small-cell lung cancer: CNS efficacy results from the ALEX study. Ann Oncol. 2018 Nov 1;29(11):2214-2222. doi: 10.1093/annonc/mdy405. PMID: 30215676; PMCID: PMC6290889.

Gridelli C, de Castro Carpeno J, Dingemans AC, Griesinger F, Grossi F, Langer C, Ohe Y, Syrigos K, Thatcher N, Das-Gupta A, Truman M, Donica M, Smoljanovic V, Bennouna J. Safety and Efficacy of Bevacizumab Plus Standard-of-Care Treatment Beyond Disease Progression in Patients With Advanced Non-Small Cell Lung Cancer: The AvaALL Randomized Clinical Trial. JAMA Oncol. 2018 Dec 1;4(12):e183486. doi: 10.1001/jamaoncol.2018.3486. Epub 2018 Dec 13. Erratum in: JAMA Oncol. 2018 Dec 1;4(12):1792. PMID: 30177994; PMCID: PMC6440713.

Wu YL, Ahn MJ, Garassino MC, Han JY, Katakami N, Kim HR, Hodge R, Kaur P, Brown AP, Ghiorghiu D, Papadimitrakopoulou VA, Mok TSK. CNS Efficacy of

Osimertinib in Patients With T790M-Positive Advanced Non-Small-Cell Lung Cancer: Data From a Randomized Phase III Trial (AURA3). J Clin Oncol. 2018 Sep 10;36(26):2702-2709. doi: 10.1200/JCO.2018.77.9363. Epub 2018 Jul 30. PMID: 30059262.

Socinski MA, Jotte RM, Cappuzzo F, Orlandi F, Stroyakovskiy D, Nogami N, Rodríguez-Abreu D, Moro-Sibilot D, Thomas CA, Barlesi F, Finley G, Kelsch C, Lee A, Coleman S, Deng Y, Shen Y, Kowanetz M, Lopez-Chavez A, Sandler A, Reck M; IMpower150 Study Group. Atezolizumab for First-Line Treatment of Metastatic Nonsquamous NSCLC. N Engl J Med. 2018 Jun 14;378(24):2288-2301. doi: 10.1056/NEJMoa1716948. Epub 2018 Jun 4. PMID: 29863955.

Lee CK, Novello S, Rydén A, Mann H, Mok T. Patient-Reported Symptoms and Impact of Treatment With Osimertinib Versus Chemotherapy in Advanced Non-Small-Cell Lung Cancer: The AURA3 Trial. J Clin Oncol. 2018 Jun 20;36(18):1853-1860. doi: 10.1200/JCO.2017.77.2293. Epub 2018 May 7. PMID: 29733770.

Gandhi L, Rodríguez-Abreu D, Gadgeel S, Esteban E, Felip E, De Angelis F, Domine M, Clingan P, Hochmair MJ, Powell SF, Cheng SY, Bischoff HG, Peled N, Grossi F, Jennens RR, Reck M, Hui R, Garon EB, Boyer M, Rubio-Viqueira B, Novello S, Kurata T, Gray JE, Vida J, Wei Z, Yang J, Raftopoulos H, Pietanza MC, Garassino MC; KEYNOTE-189 Investigators. Pembrolizumab plus Chemotherapy in Metastatic Non-Small-Cell Lung Cancer. N Engl J Med. 2018 May 31;378(22):2078-2092. doi: 10.1056/NEJMoa1801005. Epub 2018 Apr 16. PMID: 29658856.

Hellmann MD, Ciuleanu TE, Pluzanski A, Lee JS, Otterson GA, Audigier-Valette C, Minenza E, Linardou H, Burgers S, Salman P, Borghaei H, Ramalingam SS, Brahmer J, Reck M, O'Byrne KJ, Geese WJ, Green G, Chang H, Szustakowski J, Bhagavatheeswaran P, Healey D, Fu Y, Nathan F, Paz-Ares L. Nivolumab plus Ipilimumab in Lung Cancer with a High Tumor Mutational Burden. N Engl J Med. 2018 May 31;378(22):2093-2104. doi: 10.1056/NEJMoa1801946. Epub 2018 Apr 16. PMID: 29658845; PMCID: PMC7193684.

Herbst RS, Redman MW, Kim ES, Semrad TJ, Bazhenova L, Masters G, Oettel K, Guaglianone P, Reynolds C, Karnad A, Arnold SM, Varella-Garcia M, Moon J, Mack PC, Blanke CD, Hirsch FR, Kelly K, Gandara DR. Cetuximab plus carboplatin and paclitaxel with or without bevacizumab versus carboplatin and paclitaxel with or without bevacizumab in advanced NSCLC (SWOG S0819): a randomised, phase 3 study. Lancet Oncol. 2018 Jan;19(1):101-114. doi: 10.1016/S1470-2045(17)30694-0. Epub 2017 Nov 20. PMID: 29169877; PMCID: PMC5847342.

Soria JC, Ohe Y, Vansteenkiste J, Reungwetwattana T, Chewaskulyong B, Lee

KH, Dechaphunkul A, Imamura F, Nogami N, Kurata T, Okamoto I, Zhou C, Cho BC, Cheng Y, Cho EK, Voon PJ, Planchard D, Su WC, Gray JE, Lee SM, Hodge R, Marotti M, Rukazenkov Y, Ramalingam SS; FLAURA Investigators. Osimertinib in Untreated EGFR-Mutated Advanced Non-Small-Cell Lung Cancer. N Engl J Med. 2018 Jan 11;378(2):113-125. doi: 10.1056/NEJMoa1713137. Epub 2017 Nov 18. PMID: 29151359.

Ouyang X, Shi M, Jie F, Bai Y, Shen P, Yu Z, Wang X, Huang C, Tao M, Wang Z, Xie C, Wu Q, Shu Y, Han B, Zhang F, Zhang Y, Hu C, Ma X, Liang Y, Wang A, Lu B, Shi Y, Chen J, Zhuang Z, Wang J, Huang J, Wang C, Bai C, Zhou X, Li Q, Chen F, Yu H, Feng J. Phase III study of dulanermin (recombinant human tumor necrosis factor-related apoptosis-inducing ligand/Apo2 ligand) combined with vinorelbine and cisplatin in patients with advanced non-small-cell lung cancer. Invest New Drugs. 2018 Apr;36(2):315-322. doi: 10.1007/s10637-017-0536-y. Epub 2017 Nov 14. PMID: 29134432.

Reck M, Taylor F, Penrod JR, DeRosa M, Morrissey L, Dastani H, Orsini L, Gralla RJ. Impact of Nivolumab versus Docetaxel on Health-Related Quality of Life and Symptoms in Patients with Advanced Squamous Non-Small Cell Lung Cancer: Results from the CheckMate 017 Study. J Thorac Oncol. 2018 Feb;13(2):194-204. doi: 10.1016/j.jtho.2017.10.029. Epub 2017 Nov 10. PMID: 29129758.

Brahmer JR, Rodríguez-Abreu D, Robinson AG, Hui R, Csőszi T, Fülöp A, Gottfried M, Peled N, Tafreshi A, Cuffe S, O'Brien M, Rao S, Hotta K, Zhang J, Lubiniecki GM, Deitz AC, Rangwala R, Reck M. Health-related quality-of-life results for pembrolizumab versus chemotherapy in advanced, PD-L1-positive NSCLC (KEYNOTE-024): a multicentre, international, randomised, open-label phase 3 trial. Lancet Oncol. 2017 Dec;18(12):1600-1609. doi: 10.1016/S1470-2045(17)30690-3. Epub 2017 Nov 9. PMID: 29129441.

Wu YL, Cheng Y, Zhou X, Lee KH, Nakagawa K, Niho S, Tsuji F, Linke R, Rosell R, Corral J, Migliorino MR, Pluzanski A, Sbar EI, Wang T, White JL, Nadanaciva S, Sandin R, Mok TS. Dacomitinib versus gefitinib as first-line treatment for patients with EGFR-mutation-positive non-small-cell lung cancer (ARCHER 1050): a randomised, open-label, phase 3 trial. Lancet Oncol. 2017 Nov;18(11):1454-1466. doi: 10.1016/S1470-2045(17)30608-3. Epub 2017 Sep 25. PMID: 28958502.

Shi YK, Wang L, Han BH, Li W, Yu P, Liu YP, Ding CM, Song X, Ma ZY, Ren XL, Feng JF, Zhang HL, Chen GY, Han XH, Wu N, Yao C, Song Y, Zhang SC, Song W, Liu XQ, Zhao SJ, Lin YC, Ye XQ, Li K, Shu YQ, Ding LM, Tan FL, Sun Y. First-line icotinib versus cisplatin/pemetrexed plus pemetrexed maintenance therapy for patients with advanced EGFR mutation-positive lung adenocarcinoma (CONVINCE): a phase 3, open-label, randomized study. Ann Oncol. 2017 Oct 1;28(10):2443-2450. doi: 10.1093/annonc/mdx359. PMID: 28945850.

Katakami N, Felip E, Spigel DR, Kim JH, Olivo M, Guo M, Nokihara H, Yang JC, Iannotti N, Satouchi M, Barlesi F. A randomized, open-label, multicenter, phase 3 study to compare the efficacy and safety of eribulin to treatment of physician's choice in patients with advanced non-small cell lung cancer. Ann Oncol. 2017 Sep 1;28(9):2241-2247. doi: 10.1093/annonc/mdx284. PMID: 28911085; PMCID: PMC5834051.

Govindan R, Szczesna A, Ahn MJ, Schneider CP, Gonzalez Mella PF, Barlesi F, Han B, Ganea DE, Von Pawel J, Vladimirov V, Fadeeva N, Lee KH, Kurata T, Zhang L, Tamura T, Postmus PE, Jassem J, O'Byrne K, Kopit J, Li M, Tschaika M, Reck M. Phase III Trial of Ipilimumab Combined With Paclitaxel and Carboplatin in Advanced Squamous Non-Small-Cell Lung Cancer. J Clin Oncol. 2017 Oct 20;35(30):3449-3457. doi: 10.1200/JCO.2016.71.7629. Epub 2017 Aug 30. PMID: 28854067.

Ferry D, Billingham L, Jarrett H, Dunlop D, Woll PJ, Nicolson M, Shah R, Thompson J, Spicer J, Muthukumar D, Skailes G, Leonard P, Chetiyawardana AD, Wells P, Lewanski C, Crosse B, Hill M, Gaunt P, O'Byrne K. Carboplatin versus two doses of cisplatin in combination with gemcitabine in the treatment of advanced non-small-cell lung cancer: Results from a British Thoracic Oncology Group randomised phase III trial. Eur J Cancer. 2017 Sep;83:302-312. doi: 10.1016/j.ejca.2017.05.037. Epub 2017 Aug 4. PMID: 28780466; PMCID: PMC5597318.

Peters S, Camidge DR, Shaw AT, Gadgeel S, Ahn JS, Kim DW, Ou SI, Pérol M, Dziadziuszko R, Rosell R, Zeaiter A, Mitry E, Golding S, Balas B, Noe J, Morcos PN, Mok T; ALEX Trial Investigators. Alectinib versus Crizotinib in Untreated ALK-Positive Non-Small-Cell Lung Cancer. N Engl J Med. 2017 Aug 31;377(9):829-838. doi: 10.1056/NEJMoa1704795. Epub 2017 Jun 6. PMID: 28586279.

Hida T, Nokihara H, Kondo M, Kim YH, Azuma K, Seto T, Takiguchi Y, Nishio M, Yoshioka H, Imamura F, Hotta K, Watanabe S, Goto K, Satouchi M, Kozuki T, Shukuya T, Nakagawa K, Mitsudomi T, Yamamoto N, Asakawa T, Asabe R, Tanaka T, Tamura T. Alectinib versus crizotinib in patients with ALK-positive non-small-cell lung cancer (J-ALEX): an open-label, randomised phase 3 trial. Lancet. 2017 Jul 1;390(10089):29-39. doi: 10.1016/S0140-6736(17)30565-2. Epub 2017 May 10. PMID: 28501140.

Edelman MJ, Wang X, Hodgson L, Cheney RT, Baggstrom MQ, Thomas SP, Gajra A, Bertino E, Reckamp KL, Molina J, Schiller JH, Mitchell-Richards K, Friedman PN, Ritter J, Milne G, Hahn OM, Stinchcombe TE, Vokes EE; Alliance for Clinical Trials in Oncology. Phase III Randomized, Placebo-Controlled, Double-Blind Trial of Celecoxib in Addition to Standard Chemotherapy for Advanced Non-Small-Cell

Lung Cancer With Cyclooxygenase-2 Overexpression: CALGB 30801 (Alliance). J Clin Oncol. 2017 Jul 1;35(19):2184-2192. doi: 10.1200/JCO.2016.71.3743. Epub 2017 May 10. PMID: 28489511; PMCID: PMC5493050.

Seckl MJ, Ottensmeier CH, Cullen M, Schmid P, Ngai Y, Muthukumar D, Thompson J, Harden S, Middleton G, Fife KM, Crosse B, Taylor P, Nash S, Hackshaw A. Multicenter, Phase III, Randomized, Double-Blind, Placebo-Controlled Trial of Pravastatin Added to First-Line Standard Chemotherapy in Small-Cell Lung Cancer (LUNGSTAR). J Clin Oncol. 2017 May 10;35(14):1506-1514. doi: 10.1200/JCO.2016.69.7391. Epub 2017 Feb 27. PMID: 28240967; PMCID: PMC5455702.

Park CK, Oh IJ, Kim KS, Choi YD, Jang TW, Kim YS, Lee KH, Shin KC, Jung CY, Yang SH, Ryu JS, Jang SH, Yoo SS, Yong SJ, Lee KY, In KH, Lee MK, Kim YC. Randomized Phase III Study of Docetaxel Plus Cisplatin Versus Pemetrexed Plus Cisplatin as First-line Treatment of Nonsquamous Non-Small-cell Lung Cancer: A TRAIL Trial. Clin Lung Cancer. 2017 Jul;18(4):e289-e296. doi: 10.1016/j.cllc.2017.01.002. Epub 2017 Jan 11. PMID: 28185792.

Soria JC, Tan DSW, Chiari R, Wu YL, Paz-Ares L, Wolf J, Geater SL, Orlov S, Cortinovis D, Yu CJ, Hochmair M, Cortot AB, Tsai CM, Moro-Sibilot D, Campelo RG, McCulloch T, Sen P, Dugan M, Pantano S, Branle F, Massacesi C, de Castro G Jr. First-line ceritinib versus platinum-based chemotherapy in advanced ALK-rearranged non-small-cell lung cancer (ASCEND-4): a randomised, open-label, phase 3 study. Lancet. 2017 Mar 4;389(10072):917-929. doi: 10.1016/S0140-6736(17)30123-X. Epub 2017 Jan 24. Erratum in: Lancet. 2017 Mar 4;389(10072):908. PMID: 28126333.

Yang JJ, Zhou Q, Yan HH, Zhang XC, Chen HJ, Tu HY, Wang Z, Xu CR, Su J, Wang BC, Jiang BY, Bai XY, Zhong WZ, Yang XN, Wu YL. A phase III randomised controlled trial of erlotinib vs gefitinib in advanced non-small cell lung cancer with EGFR mutations. Br J Cancer. 2017 Feb 28;116(5):568-574. doi: 10.1038/bjc.2016.456. Epub 2017 Jan 19. PMID: 28103612; PMCID: PMC5344291.

Mok TS, Wu Y-L, Ahn M-J, Garassino MC, Kim HR, Ramalingam SS, Shepherd FA, He Y, Akamatsu H, Theelen WS, Lee CK, Sebastian M, Templeton A, Mann H, Marotti M, Ghiorghiu S, Papadimitrakopoulou VA; AURA3 Investigators. Osimertinib or Platinum-Pemetrexed in EGFR T790M-Positive Lung Cancer. N Engl J Med. 2017 Feb 16;376(7):629-640. doi: 10.1056/NEJMoa1612674. Epub 2016 Dec 6. PMID: 27959700; PMCID: PMC6762027.

Reck M, Rodríguez-Abreu D, Robinson AG, Hui R, Csőszi T, Fülöp A, Gottfried M, Peled N, Tafreshi A, Cuffe S, O'Brien M, Rao S, Hotta K, Leiby MA, Lubiniecki GM, Shentu Y, Rangwala R, Brahmer JR; KEYNOTE-024 Investigators. Pembrolizumab

versus Chemotherapy for PD-L1-Positive Non-Small-Cell Lung Cancer. N Engl J Med. 2016 Nov 10;375(19):1823-1833. doi: 10.1056/NEJMoa1606774. Epub 2016 Oct 8. PMID: 27718847.

Joerger M, von Pawel J, Kraff S, Fischer JR, Eberhardt W, Gauler TC, Mueller L, Reinmuth N, Reck M, Kimmich M, Mayer F, Kopp HG, Behringer DM, Ko YD, Hilger RA, Roessler M, Kloft C, Henrich A, Moritz B, Miller MC, Salamone SJ, Jaehde U. Open-label, randomized study of individualized, pharmacokinetically (PK)-guided dosing of paclitaxel combined with carboplatin or cisplatin in patients with advanced non-small-cell lung cancer (NSCLC). Ann Oncol. 2016 Oct;27(10):1895-902. doi: 10.1093/annonc/mdw290. Epub 2016 Aug 8. PMID: 27502710.

Quoix E, Lena H, Losonczy G, Forget F, Chouaid C, Papai Z, Gervais R, Ottensmeier C, Szczesna A, Kazarnowicz A, Beck JT, Westeel V, Felip E, Debieuvre D, Madroszyk A, Adam J, Lacoste G, Tavernaro A, Bastien B, Halluard C, Palanché T, Limacher JM. TG4010 immunotherapy and first-line chemotherapy for advanced non-small-cell lung cancer (TIME): results from the phase 2b part of a randomised, double-blind, placebo-controlled, phase 2b/3 trial. Lancet Oncol. 2016 Feb;17(2):212-223. doi: 10.1016/S1470-2045(15)00483-0. Epub 2015 Dec 23. PMID: 26727163.

Macbeth F, Noble S, Evans J, Ahmed S, Cohen D, Hood K, Knoyle D, Linnane S, Longo M, Moore B, Woll PJ, Appel W, Dickson J, Ferry D, Brammer C, Griffiths G. Randomized Phase III Trial of Standard Therapy Plus Low Molecular Weight Heparin in Patients With Lung Cancer: FRAGMATIC Trial. J Clin Oncol. 2016 Feb 10;34(5):488-94. doi: 10.1200/JCO.2015.64.0268. Epub 2015 Dec 23. PMID: 26700124.

BREAST CANCER

von Minckwitz G, Huang CS, Mano MS, Loibl S, Mamounas EP, Untch M, Wolmark N, Rastogi P, Schneeweiss A, Redondo A, Fischer HH, Jacot W, Conlin AK, Arce-Salinas C, Wapnir IL, Jackisch C, DiGiovanna MP, Fasching PA, Crown JP, Wülfing P, Shao Z, Rota Caremoli E, Wu H, Lam LH, Tesarowski D, Smitt M, Douthwaite H, Singel SM, Geyer CE Jr; KATHERINE Investigators. Trastuzumab Emtansine for Residual Invasive HER2-Positive Breast Cancer. N Engl J Med. 2019 Feb 14;380(7):617-628. doi: 10.1056/NEJMoa1814017. Epub 2018 Dec 5. PMID: 30516102.

Robson M, Im SA, Senkus E, Xu B, Domchek SM, Masuda N, Delaloge S, Li W, Tung N, Armstrong A, Wu W, Goessl C, Runswick S, Conte P. Olaparib for Metastatic Breast Cancer in Patients with a Germline BRCA Mutation. N Engl J Med. 2017 Aug 10;377(6):523-533. doi: 10.1056/NEJMoa1706450. Epub 2017 Jun 4. Erratum in: N

Engl J Med. 2017 Oct 26;377(17):1700. PMID: 28578601.

Hortobagyi GN, Stemmer SM, Burris HA, Yap YS, Sonke GS, Paluch-Shimon S, Campone M, Petrakova K, Blackwell KL, Winer EP, Janni W, Verma S, Conte P, Arteaga CL, Cameron DA, Mondal S, Su F, Miller M, Elmeliegy M, Germa C, O'Shaughnessy J. Updated results from MONALEESA-2, a phase III trial of first-line ribociclib plus letrozole versus placebo plus letrozole in hormone receptor-positive, HER2-negative advanced breast cancer. Ann Oncol. 2018 Jul 1;29(7):1541-1547. doi: 10.1093/annonc/mdy155. Erratum in: Ann Oncol. 2019 Nov 1;30(11):1842. PMID: 29718092.

Tutt A, Tovey H, Cheang MCU, Kernaghan S, Kilburn L, Gazinska P, Owen J, Abraham J, Barrett S, Barrett-Lee P, Brown R, Chan S, Dowsett M, Flanagan JM, Fox L, Grigoriadis A, Gutin A, Harper-Wynne C, Hatton MQ, Hoadley KA, Parikh J, Parker P, Perou CM, Roylance R, Shah V, Shaw A, Smith IE, Timms KM, Wardley AM, Wilson G, Gillett C, Lanchbury JS, Ashworth A, Rahman N, Harries M, Ellis P, Pinder SE, Bliss JM. Carboplatin in BRCA1/2-mutated and triple-negative breast cancer BRCAness subgroups: the TNT Trial. Nat Med. 2018 May;24(5):628-637. doi: 10.1038/s41591-018-0009-7. Epub 2018 Apr 30. PMID: 29713086; PMCID: PMC6372067.

Saura C, Oliveira M, Feng YH, Dai MS, Chen SW, Hurvitz SA, Kim SB, Moy B, Delaloge S, Gradishar W, Masuda N, Palacova M, Trudeau ME, Mattson J, Yap YS, Hou MF, De Laurentiis M, Yeh YM, Chang HT, Yau T, Wildiers H, Haley B, Fagnani D, Lu YS, Crown J, Lin J, Takahashi M, Takano T, Yamaguchi M, Fujii T, Yao B, Bebchuk J, Keyvanjah K, Bryce R, Brufsky A; NALA Investigators. Neratinib Plus Capecitabine Versus Lapatinib Plus Capecitabine in HER2-Positive Metastatic Breast Cancer Previously Treated With ≥ 2 HER2-Directed Regimens: Phase III NALA Trial. J Clin Oncol. 2020 Sep 20;38(27):3138-3149. doi: 10.1200/JCO.20.00147. Epub 2020 Jul 17. PMID: 32678716; PMCID: PMC7499616.

Litton JK, Rugo HS, Ettl J, Hurvitz SA, Gonçalves A, Lee KH, Fehrenbacher L, Yerushalmi R, Mina LA, Martin M, Roché H, Im YH, Quek RGW, Markova D, Tudor IC, Hannah AL, Eiermann W, Blum JL. Talazoparib in Patients with Advanced Breast Cancer and a Germline BRCA Mutation. N Engl J Med. 2018 Aug 23;379(8):753-763. doi: 10.1056/NEJMoa1802905. Epub 2018 Aug 15. PMID: 30110579.

Robson ME, Tung N, Conte P, Im SA, Senkus E, Xu B, Masuda N, Delaloge S, Li W, Armstrong A, Wu W, Goessl C, Runswick S, Domchek SM. OlympiAD final overall survival and tolerability results: Olaparib versus chemotherapy treatment of physician's choice in patients with a germline BRCA mutation and HER2-negative metastatic breast cancer. Ann Oncol. 2019 Apr 1;30(4):558-566. doi: 10.1093/annonc/mdz012. PMID: 30689707; PMCID: PMC6503629.

Swain SM, Miles D, Kim SB, Im YH, Im SA, Semiglazov V, Ciruelos E, Schneeweiss A, Loi S, Monturus E, Clark E, Knott A, Restuccia E, Benyunes MC, Cortés J; CLEOPATRA study group. Pertuzumab, trastuzumab, and docetaxel for HER2-positive metastatic breast cancer (CLEOPATRA): end-of-study results from a double-blind, randomised, placebo-controlled, phase 3 study. Lancet Oncol. 2020 Apr;21(4):519-530. doi: 10.1016/S1470-2045(19)30863-0. Epub 2020 Mar 12. PMID: 32171426.

Tripathy D, Im SA, Colleoni M, Franke F, Bardia A, Harbeck N, Hurvitz SA, Chow L, Sohn J, Lee KS, Campos-Gomez S, Villanueva Vazquez R, Jung KH, Babu KG, Wheatley-Price P, De Laurentiis M, Im YH, Kuemmel S, El-Saghir N, Liu MC, Carlson G, Hughes G, Diaz-Padilla I, Germa C, Hirawat S, Lu YS. Ribociclib plus endocrine therapy for premenopausal women with hormone-receptor-positive, advanced breast cancer (MONALEESA-7): a randomised phase 3 trial. Lancet Oncol. 2018 Jul;19(7):904-915. doi: 10.1016/S1470-2045(18)30292-4. Epub 2018 May 24. PMID: 29804902.

Winer EP, Lipatov O, Im SA, Goncalves A, Muñoz-Couselo E, Lee KS, Schmid P, Tamura K, Testa L, Witzel I, Ohtani S, Turner N, Zambelli S, Harbeck N, Andre F, Dent R, Zhou X, Karantza V, Mejia J, Cortes J; KEYNOTE-119 investigators. Pembrolizumab versus investigator-choice chemotherapy for metastatic triple-negative breast cancer (KEYNOTE-119): a randomised, open-label, phase 3 trial. Lancet Oncol. 2021 Apr;22(4):499-511. doi: 10.1016/S1470-2045(20)30754-3. Epub 2021 Mar 4. PMID: 33676601.

Hortobagyi GN, Stemmer SM, Burris HA, Yap YS, Sonke GS, Paluch-Shimon S, Campone M, Blackwell KL, André F, Winer EP, Janni W, Verma S, Conte P, Arteaga CL, Cameron DA, Petrakova K, Hart LL, Villanueva C, Chan A, Jakobsen E, Nusch A, Burdaeva O, Grischke EM, Alba E, Wist E, Marschner N, Favret AM, Yardley D, Bachelot T, Tseng LM, Blau S, Xuan F, Souami F, Miller M, Germa C, Hirawat S, O'Shaughnessy J. Ribociclib as First-Line Therapy for HR-Positive, Advanced Breast Cancer. N Engl J Med. 2016 Nov 3;375(18):1738-1748. doi: 10.1056/NEJMoa1609709. Epub 2016 Oct 7. Erratum in: N Engl J Med. 2018 Dec 27;379(26):2582. PMID: 27717303.

Rugo HS, Finn RS, Diéras V, Ettl J, Lipatov O, Joy AA, Harbeck N, Castrellon A, Iyer S, Lu DR, Mori A, Gauthier ER, Bartlett CH, Gelmon KA, Slamon DJ. Palbociclib plus letrozole as first-line therapy in estrogen receptor-positive/human epidermal growth factor receptor 2-negative advanced breast cancer with extended follow-up. Breast Cancer Res Treat. 2019 Apr;174(3):719-729. doi: 10.1007/s10549-018-05125-4. Epub 2019 Jan 10. PMID: 30632023; PMCID: PMC6438948.

Xu B, Yan M, Ma F, Hu X, Feng J, Ouyang Q, Tong Z, Li H, Zhang Q, Sun T, Wang X, Yin Y, Cheng Y, Li W, Gu Y, Chen Q, Liu J, Cheng J, Geng C, Qin S, Wang S, Lu J, Shen K, Liu Q, Wang X, Wang H, Luo T, Yang J, Wu Y, Yu Z, Zhu X, Chen C, Zou J; PHOEBE Investigators. Pyrotinib plus capecitabine versus lapatinib plus capecitabine for the treatment of HER2-positive metastatic breast cancer (PHOEBE): a multicentre, open-label, randomised, controlled, phase 3 trial. Lancet Oncol. 2021 Mar;22(3):351-360. doi: 10.1016/S1470-2045(20)30702-6. Epub 2021 Feb 11. PMID: 33581774.

Martin M, Zielinski C, Ruiz-Borrego M, Carrasco E, Turner N, Ciruelos EM, Muñoz M, Bermejo B, Margeli M, Anton A, Kahan Z, Csöszi T, Casas MI, Murillo L, Morales S, Alba E, Gal-Yam E, Guerrero-Zotano A, Calvo L, de la Haba-Rodriguez J, Ramos M, Alvarez I, Garcia-Palomo A, Huang Bartlett C, Koehler M, Caballero R, Corsaro M, Huang X, Garcia-Sáenz JA, Chacón JI, Swift C, Thallinger C, Gil-Gil M. Palbociclib in combination with endocrine therapy versus capecitabine in hormonal receptor-positive, human epidermal growth factor 2-negative, aromatase inhibitor-resistant metastatic breast cancer: a phase III randomised controlled trial-PEARL. Ann Oncol. 2021 Apr;32(4):488-499. doi: 10.1016/j.annonc.2020.12.013. Epub 2020 Dec 29. PMID: 33385521.

Chan A, Moy B, Mansi J, Ejlertsen B, Holmes FA, Chia S, Iwata H, Gnant M, Loibl S, Barrios CH, Somali I, Smichkoska S, Martinez N, Alonso MG, Link JS, Mayer IA, Cold S, Murillo SM, Senecal F, Inoue K, Ruiz-Borrego M, Hui R, Denduluri N, Patt D, Rugo HS, Johnston SRD, Bryce R, Zhang B, Xu F, Wong A, Martin M; ExteNET Study Group. Final Efficacy Results of Neratinib in HER2-positive Hormone Receptor-positive Early-stage Breast Cancer From the Phase III ExteNET Trial. Clin Breast Cancer. 2021 Feb;21(1):80-91.e7. doi: 10.1016/j.clbc.2020.09.014. Epub 2020 Oct 6. PMID: 33183970.

Loibl S, Marmé F, Martin M, Untch M, Bonnefoi H, Kim SB, Bear H, McCarthy N, Melé Olivé M, Gelmon K, García-Sáenz J, Kelly CM, Reimer T, Toi M, Rugo HS, Denkert C, Gnant M, Makris A, Koehler M, Huang-Bartelett C, Lechuga Frean MJ, Colleoni M, Werutsky G, Seiler S, Burchardi N, Nekljudova V, von Minckwitz G. Palbociclib for Residual High-Risk Invasive HR-Positive and HER2-Negative Early Breast Cancer-The Penelope-B Trial. J Clin Oncol. 2021 May 10;39(14):1518-1530. doi: 10.1200/JCO.20.03639. Epub 2021 Apr 1. PMID: 33793299.

Robertson JFR, Bondarenko IM, Trishkina E, Dvorkin M, Panasci L, Manikhas A, Shparyk Y, Cardona-Huerta S, Cheung KL, Philco-Salas MJ, Ruiz-Borrego M, Shao Z, Noguchi S, Rowbottom J, Stuart M, Grinsted LM, Fazal M, Ellis MJ. Fulvestrant 500 mg versus anastrozole 1 mg for hormone receptor-positive advanced breast cancer (FALCON): an international, randomised, double-blind, phase 3 trial. Lancet. 2016 Dec 17;388(10063):2997-3005. doi: 10.1016/S0140-6736(16)32389-3.

Epub 2016 Nov 29. PMID: 27908454.

Tan AR, Im SA, Mattar A, Colomer R, Stroyakovskii D, Nowecki Z, De Laurentiis M, Pierga JY, Jung KH, Schem C, Hogea A, Badovinac Crnjevic T, Heeson S, Shivhare M, Kirschbrown WP, Restuccia E, Jackisch C; FeDeriCa study group. Fixed-dose combination of pertuzumab and trastuzumab for subcutaneous injection plus chemotherapy in HER2-positive early breast cancer (FeDeriCa): a randomised, open-label, multicentre, non-inferiority, phase 3 study. Lancet Oncol. 2021 Jan;22(1):85-97. doi: 10.1016/S1470-2045(20)30536-2. Epub 2020 Dec 21. Erratum in: Lancet Oncol. 2021 Feb;22(2):e42. PMID: 33357420.

Mamounas EP, Bandos H, Lembersky BC, Jeong JH, Geyer CE Jr, Rastogi P, Fehrenbacher L, Graham ML, Chia SK, Brufsky AM, Walshe JM, Soori GS, Dakhil SR, Seay TE, Wade JL 3rd, McCarron EC, Paik S, Swain SM, Wickerham DL, Wolmark N. Use of letrozole after aromatase inhibitor-based therapy in postmenopausal breast cancer (NRG Oncology/NSABP B-42): a randomised, double-blind, placebo-controlled, phase 3 trial. Lancet Oncol. 2019 Jan;20(1):88-99. doi: 10.1016/S1470-2045(18)30621-1. Epub 2018 Nov 30. Erratum in: Lancet Oncol. 2019 Jan;20(1):e10. PMID: 30509771; PMCID: PMC6691732.

Litton JK, Hurvitz SA, Mina LA, Rugo HS, Lee KH, Gonçalves A, Diab S, Woodward N, Goodwin A, Yerushalmi R, Roché H, Im YH, Eiermann W, Quek RGW, Usari T, Lanzalone S, Czibere A, Blum JL, Martin M, Ettl J. Talazoparib versus chemotherapy in patients with germline BRCA1/2-mutated HER2-negative advanced breast cancer: final overall survival results from the EMBRACA trial. Ann Oncol. 2020 Nov;31(11):1526-1535. doi: 10.1016/j.annonc.2020.08.2098. Epub 2020 Aug 20. PMID: 32828825.

Bachelot T, Ciruelos E, Schneeweiss A, Puglisi F, Peretz-Yablonski T, Bondarenko I, Paluch-Shimon S, Wardley A, Merot JL, du Toit Y, Easton V, Lindegger N, Miles D; PERUSE investigators. Preliminary safety and efficacy of first-line pertuzumab combined with trastuzumab and taxane therapy for HER2-positive locally recurrent or metastatic breast cancer (PERUSE). Ann Oncol. 2019 May 1;30(5):766-773. doi: 10.1093/annonc/mdz061. PMID: 30796821.

Diéras V, Han HS, Kaufman B, Wildiers H, Friedlander M, Ayoub JP, Puhalla SL, Bondarenko I, Campone M, Jakobsen EH, Jalving M, Oprean C, Palácová M, Park YH, Shparyk Y, Yañez E, Khandelwal N, Kundu MG, Dudley M, Ratajczak CK, Maag D, Arun BK. Veliparib with carboplatin and paclitaxel in BRCA-mutated advanced breast cancer (BROCADE3): a randomised, double-blind, placebo-controlled, phase 3 trial. Lancet Oncol. 2020 Oct;21(10):1269-1282. doi: 10.1016/S1470-2045(20)30447-2. Epub 2020 Aug 27. PMID: 32861273.

Connolly RM, Zhao F, Miller KD, Lee MJ, Piekarz RL, Smith KL, Brown-Glaberman UA, Winn JS, Faller BA, Onitilo AA, Burkard ME, Budd GT, Levine EG, Royce ME, Kaufman PA, Thomas A, Trepel JB, Wolff AC, Sparano JA. E2112: Randomized Phase III Trial of Endocrine Therapy Plus Entinostat or Placebo in Hormone Receptor-Positive Advanced Breast Cancer. A Trial of the ECOG-ACRIN Cancer Research Group. J Clin Oncol. 2021 Oct 1;39(28):3171-3181. doi: 10.1200/JCO.21.00944. Epub 2021 Aug 6. PMID: 34357781; PMCID: PMC8478386.

Perez EA, Barrios C, Eiermann W, Toi M, Im YH, Conte P, Martin M, Pienkowski T, Pivot X, Burris H 3rd, Petersen JA, Stanzel S, Strasak A, Patre M, Ellis P. Trastuzumab Emtansine With or Without Pertuzumab Versus Trastuzumab Plus Taxane for Human Epidermal Growth Factor Receptor 2-Positive, Advanced Breast Cancer: Primary Results From the Phase III MARIANNE Study. J Clin Oncol. 2017 Jan 10;35(2):141-148. doi: 10.1200/JCO.2016.67.4887. Epub 2016 Nov 7. Erratum in: J Clin Oncol. 2017 Jul 10;35(20):2342. Erratum in: J Clin Oncol. 2019 Feb 1;37(4):358. PMID: 28056202; PMCID: PMC5455677.

Xu B, Zhang Q, Zhang P, Hu X, Li W, Tong Z, Sun T, Teng Y, Wu X, Ouyang Q, Yan X, Cheng J, Liu Q, Feng J, Wang X, Yin Y, Shi Y, Pan Y, Wang Y, Xie W, Yan M, Liu Y, Yan P, Wu F, Zhu X, Zou J; DAWNA-1 Study Consortium. Dalpiciclib or placebo plus fulvestrant in hormone receptor-positive and HER2-negative advanced breast cancer: a randomized, phase 3 trial. Nat Med. 2021 Nov;27(11):1904-1909. doi: 10.1038/s41591-021-01562-9. Epub 2021 Nov 4. PMID: 34737452.

Bardia A, Aftimos P, Bihani T, Anderson-Villaluz AT, Jung J, Conlan MG, Kaklamani VG. EMERALD: Phase III trial of elacestrant (RAD1901) vs endocrine therapy for previously treated ER+ advanced breast cancer. Future Oncol. 2019 Oct;15(28):3209-3218. doi: 10.2217/fon-2019-0370. Epub 2019 Aug 20. PMID: 31426673.

Dent S, Cortés J, Im YH, Diéras V, Harbeck N, Krop IE, Wilson TR, Cui N, Schimmoller F, Hsu JY, He J, De Laurentiis M, Sousa S, Drullinsky P, Jacot W. Phase III randomized study of taselisib or placebo with fulvestrant in estrogen receptor-positive, PIK3CA-mutant, HER2-negative, advanced breast cancer: the SANDPIPER trial. Ann Oncol. 2021 Feb;32(2):197-207. doi: 10.1016/j.annonc.2020.10.596. Epub 2020 Nov 10. PMID: 33186740; PMCID: PMC8457522.

Johnston SRD, Hegg R, Im SA, Park IH, Burdaeva O, Kurteva G, Press MF, Tjulandin S, Iwata H, Simon SD, Kenny S, Sarp S, Izquierdo MA, Williams LS, Gradishar WJ. Phase III, Randomized Study of Dual Human Epidermal Growth Factor Receptor 2 (HER2) Blockade With Lapatinib Plus Trastuzumab in Combination With an Aromatase Inhibitor in Postmenopausal Women With HER2-Positive, Hormone

Receptor-Positive Metastatic Breast Cancer: Updated Results of ALTERNATIVE. J Clin Oncol. 2021 Jan 1;39(1):79-89. doi: 10.1200/JCO.20.01894. Epub 2020 Aug 21. PMID: 32822287.

De Laurentiis M, Borstnar S, Campone M, Warner E, Bofill JS, Jacot W, Dent S, Martin M, Ring A, Cottu P, Lu J, Ciruelos E, Azim HA, Chatterjee S, Zhou K, Wu J, Menon-Singh L, Zamagni C. Full population results from the core phase of CompLEEment-1, a phase 3b study of ribociclib plus letrozole as first-line therapy for advanced breast cancer in an expanded population. Breast Cancer Res Treat. 2021 Oct;189(3):689-699. doi: 10.1007/s10549-021-06334-0. Epub 2021 Aug 19. Erratum in: Breast Cancer Res Treat. 2021 Oct 8;: PMID: 34414532; PMCID: PMC8505291.

Krop IE, Kim SB, Martin AG, LoRusso PM, Ferrero JM, Badovinac-Crnjevic T, Hoersch S, Smitt M, Wildiers H. Trastuzumab emtansine versus treatment of physician's choice in patients with previously treated HER2-positive metastatic breast cancer (TH3RESA): final overall survival results from a randomised open-label phase 3 trial. Lancet Oncol. 2017 Jun;18(6):743-754. doi: 10.1016/S1470-2045(17)30313-3. Epub 2017 May 16. PMID: 28526538.

Jiang Z, Li W, Hu X, Zhang Q, Sun T, Cui S, Wang S, Ouyang Q, Yin Y, Geng C, Tong Z, Cheng Y, Pan Y, Sun Y, Wang H, Ouyang T, Gu K, Feng J, Wang X, Wang S, Liu T, Gao J, Cristofanilli M, Ning Z, Lu X. Tucidinostat plus exemestane for postmenopausal patients with advanced, hormone receptor-positive breast cancer (ACE): a randomised, double-blind, placebo-controlled, phase 3 trial. Lancet Oncol. 2019 Jun;20(6):806-815. doi: 10.1016/S1470-2045(19)30164-0. Epub 2019 Apr 27. PMID: 31036468.

Stebbing J, Baranau Y, Baryash V, Manikhas A, Moiseyenko V, Dzagnidze G, Zhavrid E, Boliukh D, Stroyakovskii D, Pikiel J, Eniu A, Komov D, Morar-Bolba G, Li RK, Rusyn A, Lee SJ, Lee SY, Esteva FJ. CT-P6 compared with reference trastuzumab for HER2-positive breast cancer: a randomised, double-blind, active-controlled, phase 3 equivalence trial. Lancet Oncol. 2017 Jul;18(7):917-928. doi: 10.1016/S1470-2045(17)30434-5. Epub 2017 Jun 4. Erratum in: Lancet Oncol. 2017 Aug;18(8):e433. Erratum in: Lancet Oncol. 2017 Sep;18(9):e510. PMID: 28592386.

Verma S, Bartlett CH, Schnell P, DeMichele AM, Loi S, Ro J, Colleoni M, Iwata H, Harbeck N, Cristofanilli M, Zhang K, Thiele A, Turner NC, Rugo HS. Palbociclib in Combination With Fulvestrant in Women With Hormone Receptor-Positive/HER2-Negative Advanced Metastatic Breast Cancer: Detailed Safety Analysis From a Multicenter, Randomized, Placebo-Controlled, Phase III Study (PALOMA-3). Oncologist. 2016 Oct;21(10):1165-1175. doi:

10.1634/theoncologist.2016-0097. Epub 2016 Jul 1. PMID: 27368881; PMCID: PMC5061543.

Baselga J, Im SA, Iwata H, Cortés J, De Laurentiis M, Jiang Z, Arteaga CL, Jonat W, Clemons M, Ito Y, Awada A, Chia S, Jagiełło-Gruszfeld A, Pistilli B, Tseng LM, Hurvitz S, Masuda N, Takahashi M, Vuylsteke P, Hachemi S, Dharan B, Di Tomaso E, Urban P, Massacesi C, Campone M. Buparlisib plus fulvestrant versus placebo plus fulvestrant in postmenopausal, hormone receptor-positive, HER2-negative, advanced breast cancer (BELLE-2): a randomised, double-blind, placebo-controlled, phase 3 trial. Lancet Oncol. 2017 Jul;18(7):904-916. doi: 10.1016/S1470-2045(17)30376-5. Epub 2017 May 30. Erratum in: Lancet Oncol. 2019 Feb;20(2):e71-e72. PMID: 28576675; PMCID: PMC5549667.

Xu B, Zhang Q, Sun T, Li W, Teng Y, Hu X, Bondarenko I, Adamchuk H, Zhang L, Trukhin D, Wang S, Zheng H, Tong Z, Shparyk Y, Wang Q; HLX02-BC01 Investigators. Efficacy, Safety, and Immunogenicity of HLX02 Compared with Reference Trastuzumab in Patients with Recurrent or Metastatic HER2-Positive Breast Cancer: A Randomized Phase III Equivalence Trial. BioDrugs. 2021 May;35(3):337-350. doi: 10.1007/s40259-021-00475-w. Epub 2021 Apr 7. PMID: 33826080; PMCID: PMC8084805.

Shimomura A, Tamura K, Tanaka K, Mizutani T, Sasaki K, Sekino Y, Sawaki M, Shien T, Shibata T, Iwata H. A randomized phase III study comparing trastuzumab emtansine with trastuzumab, pertuzumab and docetaxel in elderly patients with advanced stage HER2-positive breast cancer: Japan Clinical Oncology Group Study (JCOG1607, HERB TEA study). Jpn J Clin Oncol. 2021 Aug 30;51(9):1471-1474. doi: 10.1093/jjco/hyab101. Erratum in: Jpn J Clin Oncol. 2021 Jul 23;: PMID: 34215870.

Ciruelos EM, Montaño A, Rodríguez CA, González-Flores E, Lluch A, Garrigós L, Quiroga V, Antón A, Malón D, Chacón JI, Velasco M, Gonzalez-Cortijo L, Jolis L, Echarri MJ, Muñoz M, Pascual T, Amigo Y, Casas M, Carrasco E, Casas A. Phase III study to evaluate patient's preference of subcutaneous versus intravenous trastuzumab in HER2-positive metastatic breast cancer patients: Results from the ChangHER study (GEICAM/2012-07). Eur J Cancer Care (Engl). 2020 Jul;29(4):e13253. doi: 10.1111/ecc.13253. Epub 2020 Jun 23. PMID: 32578279.

Tripathy D, Tolaney SM, Seidman AD, Anders CK, Ibrahim N, Rugo HS, Twelves C, Dieras V, Müller V, Tagliaferri M, Hannah AL, Cortés J. ATTAIN: Phase III study of etirinotecan pegol versus treatment of physician's choice in patients with metastatic breast cancer and brain metastases. Future Oncol. 2019 Jul;15(19):2211-2225. doi: 10.2217/fon-2019-0180. Epub 2019 May 10. PMID:

31074641; PMCID: PMC7466911.

Woodward N, De Boer RH, Redfern A, White M, Young J, Truman M, Beith J. Results From the First Multicenter, Open-label, Phase IIIb Study Investigating the Combination of Pertuzumab With Subcutaneous Trastuzumab and a Taxane in Patients With HER2-positive Metastatic Breast Cancer (SAPPHIRE). Clin Breast Cancer. 2019 Jun;19(3):216-224. doi: 10.1016/j.clbc.2019.02.008. Epub 2019 Feb 27. PMID: 30922805.

Tesch H, Stoetzer O, Decker T, Kurbacher CM, Marmé F, Schneeweiss A, Mundhenke C, Distelrath A, Fasching PA, Lux MP, Lüftner D, Hadji P, Janni W, Muth M, Kreuzeder J, Quiering C, Taran FA. Efficacy and safety of everolimus plus exemestane in postmenopausal women with hormone receptor-positive, human epidermal growth factor receptor 2-negative locally advanced or metastatic breast cancer: Results of the single-arm, phase IIIB 4EVER trial. Int J Cancer. 2019 Feb 15;144(4):877-885. doi: 10.1002/ijc.31738. Epub 2018 Oct 30. PMID: 29992557; PMCID: PMC6587781.

Miles D, Cameron D, Hilton M, Garcia J, O'Shaughnessy J. Overall survival in MERiDiAN, a double-blind placebo-controlled randomised phase III trial evaluating first-line bevacizumab plus paclitaxel for HER2-negative metastatic breast cancer. Eur J Cancer. 2018 Feb;90:153-155. doi: 10.1016/j.ejca.2017.10.018. Epub 2017 Nov 23. PMID: 29174181.

Campone M, Im SA, Iwata H, Clemons M, Ito Y, Awada A, Chia S, Jagiełło-Gruszfeld A, Pistilli B, Tseng LM, Hurvitz S, Masuda N, Cortés J, De Laurentiis M, Arteaga CL, Jiang Z, Jonat W, Le Mouhaër S, Sankaran B, Bourdeau L, El-Hashimy M, Sellami D, Baselga J. Buparlisib plus fulvestrant versus placebo plus fulvestrant for postmenopausal, hormone receptor-positive, human epidermal growth factor receptor 2-negative, advanced breast cancer: Overall survival results from BELLE-2. Eur J Cancer. 2018 Nov;103:147-154. doi: 10.1016/j.ejca.2018.08.002. Epub 2018 Sep 18. PMID: 30241001.

Di Leo A, Johnston S, Lee KS, Ciruelos E, Lønning PE, Janni W, O'Regan R, Mouret-Reynier MA, Kalev D, Egle D, Csőszi T, Bordonaro R, Decker T, Tjan-Heijnen VCG, Blau S, Schirone A, Weber D, El-Hashimy M, Dharan B, Sellami D, Bachelot T. Buparlisib plus fulvestrant in postmenopausal women with hormone-receptor-positive, HER2-negative, advanced breast cancer progressing on or after mTOR inhibition (BELLE-3): a randomised, double-blind, placebo-controlled, phase 3 trial. Lancet Oncol. 2018 Jan;19(1):87-100. doi: 10.1016/S1470-2045(17)30688-5. Epub 2017 Dec 7. Erratum in: Lancet Oncol. 2018 Mar;19(3):e137. PMID: 29223745.

Baselga J, Zamagni C, Gómez P, Bermejo B, Nagai SE, Melichar B, Chan A, Mángel L, Bergh J, Costa F, Gómez HL, Gradishar WJ, Hudis CA, Rapoport BL, Roché H, Maeda P, Huang L, Meinhardt G, Zhang J, Schwartzberg LS. RESILIENCE: Phase III Randomized, Double-Blind Trial Comparing Sorafenib With Capecitabine Versus Placebo With Capecitabine in Locally Advanced or Metastatic HER2-Negative Breast Cancer. Clin Breast Cancer. 2017 Dec;17(8):585-594.e4. doi: 10.1016/j.clbc.2017.05.006. Epub 2017 May 22. PMID: 28830796; PMCID: PMC5699974.

Martín M, Chan A, Dirix L, O'Shaughnessy J, Hegg R, Manikhas A, Shtivelband M, Krivorotko P, Batista López N, Campone M, Ruiz Borrego M, Khan QJ, Beck JT, Ramos Vázquez M, Urban P, Goteti S, Di Tomaso E, Massacesi C, Delaloge S. A randomized adaptive phase II/III study of buparlisib, a pan-class I PI3K inhibitor, combined with paclitaxel for the treatment of HER2- advanced breast cancer (BELLE-4). Ann Oncol. 2017 Feb 1;28(2):313-320. doi: 10.1093/annonc/mdw562. PMID: 27803006.

Noguchi S, Kim HJ, Jesena A, Parmar V, Sato N, Wang HC, Lokejaroenlarb S, Isidro J, Kim KS, Itoh Y, Shin E. Phase 3, open-label, randomized study comparing 3-monthly with monthly goserelin in pre-menopausal women with estrogen receptor-positive advanced breast cancer. Breast Cancer. 2016 Sep;23(5):771-9. doi: 10.1007/s12282-015-0637-4. Epub 2015 Sep 9. PMID: 26350351; PMCID: PMC4999470.

Rochlitz C, Bigler M, von Moos R, Bernhard J, Matter-Walstra K, Wicki A, Zaman K, Anchisi S, Küng M, Na KJ, Bärtschi D, Borner M, Rordorf T, Rauch D, Müller A, Ruhstaller T, Vetter M, Trojan A, Hasler-Strub U, Cathomas R, Winterhalder R; Swiss Group for Clinical Cancer Research (SAKK). SAKK 24/09: safety and tolerability of bevacizumab plus paclitaxel vs. bevacizumab plus metronomic cyclophosphamide and capecitabine as first-line therapy in patients with HER2-negative advanced stage breast cancer - a multicenter, randomized phase III trial. BMC Cancer. 2016 Oct 10;16(1):780. doi: 10.1186/s12885-016-2823-y. PMID: 27724870; PMCID: PMC5057418.

Miles D, Cameron D, Bondarenko I, Manzyuk L, Alcedo JC, Lopez RI, Im SA, Canon JL, Shparyk Y, Yardley DA, Masuda N, Ro J, Denduluri N, Hubeaux S, Quah C, Bais C, O'Shaughnessy J. Bevacizumab plus paclitaxel versus placebo plus paclitaxel as first-line therapy for HER2-negative metastatic breast cancer (MERiDiAN): A double-blind placebo-controlled randomised phase III trial with prospective biomarker evaluation. Eur J Cancer. 2017 Jan;70:146-155. doi: 10.1016/j.ejca.2016.09.024. Epub 2016 Nov 4. PMID: 27817944.

Harbeck N, Saupe S, Jäger E, Schmidt M, Kreienberg R, Müller L, Otremba BJ, Waldenmaier D, Dorn J, Warm M, Scholz M, Untch M, de Wit M, Barinoff J, Lück HJ,

Harter P, Augustin D, Harnett P, Beckmann MW, Al-Batran SE; PELICAN Investigators. A randomized phase III study evaluating pegylated liposomal doxorubicin versus capecitabine as first-line therapy for metastatic breast cancer: results of the PELICAN study. Breast Cancer Res Treat. 2017 Jan;161(1):63-72. doi: 10.1007/s10549-016-4033-3. Epub 2016 Oct 31. PMID: 27798749; PMCID: PMC5222915.

Apsangikar P, Chaudhry S, Naik M, Deoghare S, Joseph J. A comparative phase III clinical study to evaluate efficacy and safety of TrastuRel™ (biosimilar trastuzumab) and innovator trastuzumab in patients with metastatic human epidermal growth factor receptor 2 (HER2)-overexpressing breast cancer. Indian J Cancer. 2017 Oct-Dec;54(4):664-668. doi: 10.4103/ijc.IJC_449_17. PMID: 30082554.

Welt A, Marschner N, Lerchenmueller C, Decker T, Steffens CC, Koehler A, Depenbusch R, Busies S, Hegewisch-Becker S. Capecitabine and bevacizumab with or without vinorelbine in first-line treatment of HER2/neu-negative metastatic or locally advanced breast cancer: final efficacy and safety data of the randomised, open-label superiority phase 3 CARIN trial. Breast Cancer Res Treat. 2016 Feb;156(1):97-107. doi: 10.1007/s10549-016-3727-x. Epub 2016 Feb 29. PMID: 26927446; PMCID: PMC4788680.

Jain MM, Gupte SU, Patil SG, Pathak AB, Deshmukh CD, Bhatt N, Haritha C, Govind Babu K, Bondarde SA, Digumarti R, Bajpai J, Kumar R, Bakshi AV, Bhattacharya GS, Patil P, Subramanian S, Vaid AK, Desai CJ, Khopade A, Chimote G, Bapsy PP, Bhowmik S. Paclitaxel injection concentrate for nanodispersion versus nab-paclitaxel in women with metastatic breast cancer: a multicenter, randomized, comparative phase II/III study. Breast Cancer Res Treat. 2016 Feb;156(1):125-34. doi: 10.1007/s10549-016-3736-9. Epub 2016 Mar 3. PMID: 26941199; PMCID: PMC4788678.

Park IH, Sohn JH, Kim SB, Lee KS, Chung JS, Lee SH, Kim TY, Jung KH, Cho EK, Kim YS, Song HS, Seo JH, Ryoo HM, Lee SA, Yoon SY, Kim CS, Kim YT, Kim SY, Jin MR, Ro J. An Open-Label, Randomized, Parallel, Phase III Trial Evaluating the Efficacy and Safety of Polymeric Micelle-Formulated Paclitaxel Compared to Conventional Cremophor EL-Based Paclitaxel for Recurrent or Metastatic HER2-Negative Breast Cancer. Cancer Res Treat. 2017 Jul;49(3):569-577. doi: 10.4143/crt.2016.289. Epub 2016 Sep 12. PMID: 27618821; PMCID: PMC5512366.

Zielinski C, Láng I, Inbar M, Kahán Z, Greil R, Beslija S, Stemmer SM, Zvirbule Z, Steger GG, Melichar B, Pienkowski T, Sirbu D, Petruzelka L, Eniu A, Nisenbaum B, Dank M, Anghel R, Messinger D, Brodowicz T; TURANDOT investigators. Bevacizumab plus paclitaxel versus bevacizumab plus capecitabine as first-line

treatment for HER2-negative metastatic breast cancer (TURANDOT): primary endpoint results of a randomised, open-label, non-inferiority, phase 3 trial. Lancet Oncol. 2016 Sep;17(9):1230-9. doi: 10.1016/S1470-2045(16)30154-1. Epub 2016 Aug 5. PMID: 27501767.

Takashima T, Mukai H, Hara F, Matsubara N, Saito T, Takano T, Park Y, Toyama T, Hozumi Y, Tsurutani J, Imoto S, Watanabe T, Sagara Y, Nishimura R, Shimozuma K, Ohashi Y; SELECT BC Study Group. Taxanes versus S-1 as the first-line chemotherapy for metastatic breast cancer (SELECT BC): an open-label, non-inferiority, randomised phase 3 trial. Lancet Oncol. 2016 Jan;17(1):90-8. doi: 10.1016/S1470-2045(15)00411-8. Epub 2015 Nov 27. PMID: 26617202.

Slamon DJ, Neven P, Chia S, Fasching PA, De Laurentiis M, Im SA, Petrakova K, Bianchi GV, Esteva FJ, Martín M, Nusch A, Sonke GS, De la Cruz-Merino L, Beck JT, Pivot X, Sondhi M, Wang Y, Chakravartty A, Rodriguez-Lorenc K, Taran T, Jerusalem G. Overall Survival with Ribociclib plus Fulvestrant in Advanced Breast Cancer. N Engl J Med. 2020 Feb 6;382(6):514-524. doi: 10.1056/NEJMoa1911149. Epub 2019 Dec 11. PMID: 31826360.

Schmid P, Adams S, Rugo HS, Schneeweiss A, Barrios CH, Iwata H, Diéras V, Hegg R, Im SA, Shaw Wright G, Henschel V, Molinero L, Chui SY, Funke R, Husain A, Winer EP, Loi S, Emens LA; IMpassion130 Trial Investigators. Atezolizumab and Nab-Paclitaxel in Advanced Triple-Negative Breast Cancer. N Engl J Med. 2018 Nov 29;379(22):2108-2121. doi: 10.1056/NEJMoa1809615. Epub 2018 Oct 20. PMID: 30345906.

Cortes J, Cescon DW, Rugo HS, Nowecki Z, Im SA, Yusof MM, Gallardo C, Lipatov O, Barrios CH, Holgado E, Iwata H, Masuda N, Otero MT, Gokmen E, Loi S, Guo Z, Zhao J, Aktan G, Karantza V, Schmid P; KEYNOTE-355 Investigators. Pembrolizumab plus chemotherapy versus placebo plus chemotherapy for previously untreated locally recurrent inoperable or metastatic triple-negative breast cancer (KEYNOTE-355): a randomised, placebo-controlled, double-blind, phase 3 clinical trial. Lancet. 2020 Dec 5;396(10265):1817-1828. doi: 10.1016/S0140-6736(20)32531-9. PMID: 33278935.

Schmid P, Rugo HS, Adams S, Schneeweiss A, Barrios CH, Iwata H, Diéras V, Henschel V, Molinero L, Chui SY, Maiya V, Husain A, Winer EP, Loi S, Emens LA; IMpassion130 Investigators. Atezolizumab plus nab-paclitaxel as first-line treatment for unresectable, locally advanced or metastatic triple-negative breast cancer (IMpassion130): updated efficacy results from a randomised, double-blind, placebo-controlled, phase 3 trial. Lancet Oncol. 2020 Jan;21(1):44-59. doi: 10.1016/S1470-2045(19)30689-8. Epub 2019 Nov 27. PMID: 31786121.

Slamon DJ, Neven P, Chia S, Fasching PA, De Laurentiis M, Im SA, Petrakova K, Bianchi GV, Esteva FJ, Martín M, Nusch A, Sonke GS, De la Cruz-Merino L, Beck JT, Pivot X, Vidam G, Wang Y, Rodriguez Lorenc K, Miller M, Taran T, Jerusalem G. Phase III Randomized Study of Ribociclib and Fulvestrant in Hormone Receptor-Positive, Human Epidermal Growth Factor Receptor 2-Negative Advanced Breast Cancer: MONALEESA-3. J Clin Oncol. 2018 Aug 20;36(24):2465-2472. doi: 10.1200/JCO.2018.78.9909. Epub 2018 Jun 3. PMID: 29860922.

Im SA, Lu YS, Bardia A, Harbeck N, Colleoni M, Franke F, Chow L, Sohn J, Lee KS, Campos-Gomez S, Villanueva-Vazquez R, Jung KH, Chakravartty A, Hughes G, Gounaris I, Rodriguez-Lorenc K, Taran T, Hurvitz S, Tripathy D. Overall Survival with Ribociclib plus Endocrine Therapy in Breast Cancer. N Engl J Med. 2019 Jul 25;381(4):307-316. doi: 10.1056/NEJMoa1903765. Epub 2019 Jun 4. PMID: 31166679.

Miles D, Ciruelos E, Schneeweiss A, Puglisi F, Peretz-Yablonski T, Campone M, Bondarenko I, Nowecki Z, Errihani H, Paluch-Shimon S, Wardley A, Merot JL, Trask P, du Toit Y, Pena-Murillo C, Revelant V, Klingbiel D, Bachelot T; PERUSE investigators. Final results from the PERUSE study of first-line pertuzumab plus trastuzumab plus a taxane for HER2-positive locally recurrent or metastatic breast cancer, with a multivariable approach to guide prognostication. Ann Oncol. 2021 Oct;32(10):1245-1255. doi: 10.1016/j.annonc.2021.06.024. Epub 2021 Jul 2. PMID: 34224826.

Montemurro F, Delaloge S, Barrios CH, Wuerstlein R, Anton A, Brain E, Hatschek T, Kelly CM, Peña-Murillo C, Yilmaz M, Donica M, Ellis P. Trastuzumab emtansine (T-DM1) in patients with HER2-positive metastatic breast cancer and brain metastases: exploratory final analysis of cohort 1 from KAMILLA, a single-arm phase IIIb clinical trial[☆]. Ann Oncol. 2020 Oct;31(10):1350-1358. doi: 10.1016/j.annonc.2020.06.020. Epub 2020 Jul 5. PMID: 32634611.

Slamon DJ, Neven P, Chia S, Jerusalem G, De Laurentiis M, Im S, Petrakova K, Valeria Bianchi G, Martín M, Nusch A, Sonke GS, De la Cruz-Merino L, Beck JT, Ji Y, Wang C, Deore U, Chakravartty A, Zarate JP, Taran T, Fasching PA. Ribociclib plus fulvestrant for postmenopausal women with hormone receptor-positive, human epidermal growth factor receptor 2-negative advanced breast cancer in the phase III randomized MONALEESA-3 trial: updated overall survival. Ann Oncol. 2021 Aug;32(8):1015-1024. doi: 10.1016/j.annonc.2021.05.353. Epub 2021 Jun 5. Erratum in: Ann Oncol. 2021 Oct;32(10):1307. PMID: 34102253.

Goetz MP, Toi M, Campone M, Sohn J, Paluch-Shimon S, Huober J, Park IH, Trédan O, Chen SC, Manso L, Freedman OC, Garnica Jaliffe G, Forrester T, Frenzel M, Barriga S, Smith IC, Bourayou N, Di Leo A. MONARCH 3: Abemaciclib As Initial

Therapy for Advanced Breast Cancer. J Clin Oncol. 2017 Nov 10;35(32):3638-3646. doi: 10.1200/JCO.2017.75.6155. Epub 2017 Oct 2. PMID: 28968163.

Yardley DA, Coleman R, Conte P, Cortes J, Brufsky A, Shtivelband M, Young R, Bengala C, Ali H, Eakel J, Schneeweiss A, de la Cruz-Merino L, Wilks S, O'Shaughnessy J, Glück S, Li H, Miller J, Barton D, Harbeck N; tnAcity investigators. nab-Paclitaxel plus carboplatin or gemcitabine versus gemcitabine plus carboplatin as first-line treatment of patients with triple-negative metastatic breast cancer: results from the tnAcity trial. Ann Oncol. 2018 Aug 1;29(8):1763-1770. doi: 10.1093/annonc/mdy201. PMID: 29878040; PMCID: PMC6096741.

Kuemmel S, Tondini CA, Abraham J, Nowecki Z, Itrych B, Hitre E, Karaszewska B, Juárez-Ramiro A, Morales-Vásquez F, Pérez-García JM, Cardona-Huerta S, Monturus E, Sequi M, Restuccia E, Benyunes M, Martín M. Subcutaneous trastuzumab with pertuzumab and docetaxel in HER2-positive metastatic breast cancer: Final analysis of MetaPHER, a phase IIIb single-arm safety study. Breast Cancer Res Treat. 2021 Jun;187(2):467-476. doi: 10.1007/s10549-021-06145-3. Epub 2021 Mar 21. PMID: 33748921; PMCID: PMC8189949.

Schmid P, Cortes J, Pusztai L, McArthur H, Kümmel S, Bergh J, Denkert C, Park YH, Hui R, Harbeck N, Takahashi M, Foukakis T, Fasching PA, Cardoso F, Untch M, Jia L, Karantza V, Zhao J, Aktan G, Dent R, O'Shaughnessy J; KEYNOTE-522 Investigators. Pembrolizumab for Early Triple-Negative Breast Cancer. N Engl J Med. 2020 Feb 27;382(9):810-821. doi: 10.1056/NEJMoa1910549. PMID: 32101663.

Miles D, Gligorov J, André F, Cameron D, Schneeweiss A, Barrios C, Xu B, Wardley A, Kaen D, Andrade L, Semiglazov V, Reinisch M, Patel S, Patre M, Morales L, Patel SL, Kaul M, Barata T, O'Shaughnessy J; IMpassion131 investigators. Primary results from IMpassion131, a double-blind, placebo-controlled, randomised phase III trial of first-line paclitaxel with or without atezolizumab for unresectable locally advanced/metastatic triple-negative breast cancer. Ann Oncol. 2021 Aug;32(8):994-1004. doi: 10.1016/j.annonc.2021.05.801. Epub 2021 Jul 1. PMID: 34219000.

Bardia A, Hurvitz SA, Tolaney SM, Loirat D, Punie K, Oliveira M, Brufsky A, Sardesai SD, Kalinsky K, Zelnak AB, Weaver R, Traina T, Dalenc F, Aftimos P, Lynce F, Diab S, Cortés J, O'Shaughnessy J, Diéras V, Ferrario C, Schmid P, Carey LA, Gianni L, Piccart MJ, Loibl S, Goldenberg DM, Hong Q, Olivo MS, Itri LM, Rugo HS; ASCENT Clinical Trial Investigators. Sacituzumab Govitecan in Metastatic Triple-Negative Breast Cancer. N Engl J Med. 2021 Apr 22;384(16):1529-1541. doi: 10.1056/NEJMoa2028485. PMID: 33882206.

Finn RS, Martin M, Rugo HS, Jones S, Im SA, Gelmon K, Harbeck N, Lipatov ON, Walshe JM, Moulder S, Gauthier E, Lu DR, Randolph S, Diéras V, Slamon DJ. Palbociclib and Letrozole in Advanced Breast Cancer. N Engl J Med. 2016 Nov 17;375(20):1925-1936. doi: 10.1056/NEJMoa1607303. PMID: 27959613.

Dickler MN, Barry WT, Cirrincione CT, Ellis MJ, Moynahan ME, Innocenti F, Hurria A, Rugo HS, Lake DE, Hahn O, Schneider BP, Tripathy D, Carey LA, Winer EP, Hudis CA. Phase III Trial Evaluating Letrozole As First-Line Endocrine Therapy With or Without Bevacizumab for the Treatment of Postmenopausal Women With Hormone Receptor-Positive Advanced-Stage Breast Cancer: CALGB 40503 (Alliance). J Clin Oncol. 2016 Aug 1;34(22):2602-9. doi: 10.1200/JCO.2015.66.1595. Epub 2016 May 2. PMID: 27138575; PMCID: PMC5012690.

COLORECTAL CANCER

Kim TW, Taieb J, Gurary EB, Lerman N, Cui K, Yoshino T. Olaparib with or without bevacizumab or bevacizumab and 5-fluorouracil in advanced colorectal cancer: Phase III LYNK-003. Future Oncol. 2021 Dec;17(36):5013-5022. doi: 10.2217/fon-2021-0899. Epub 2021 Nov 15. PMID: 34779646.

Denda T, Takashima A, Gamoh M, Iwanaga I, Komatsu Y, Takahashi M, Nakamura M, Ohori H, Sakashita A, Tsuda M, Kobayashi Y, Baba H, Kotake M, Ishioka C, Yamada Y, Sato A, Yuki S, Morita S, Takahashi S, Yamaguchi T, Shimada K. Combination therapy of bevacizumab with either S-1 and irinotecan or mFOLFOX6/CapeOX as first-line treatment of metastatic colorectal cancer (TRICOLORE): Exploratory analysis of RAS status and primary tumour location in a randomised, open-label, phase III, non-inferiority trial. Eur J Cancer. 2021 Sep;154:296-306. doi: 10.1016/j.ejca.2021.06.013. Epub 2021 Jul 22. PMID: 34304054.

Qin S, Li J, Bai Y, Shu Y, Li W, Yin X, Cheng Y, Sun G, Deng Y, Zhong H, Li Y, Qian X, Zhang L, Zhang J, Chen K, Kang W; HLX04-mCRC03 Investigators. Efficacy, Safety, and Immunogenicity of HLX04 Versus Reference Bevacizumab in Combination with XELOX or mFOLFOX6 as First-Line Treatment for Metastatic Colorectal Cancer: Results of a Randomized, Double-Blind Phase III Study. BioDrugs. 2021 Jul;35(4):445-458. doi: 10.1007/s40259-021-00484-9. Epub 2021 May 20. PMID: 34014555; PMCID: PMC8295119.

Dasari A, Sobrero A, Yao J, Yoshino T, Schelman W, Yang Z, Chien C, Kania M, Tabernero J, Eng C. FRESCO-2: a global Phase III study investigating the efficacy and safety of fruquintinib in metastatic colorectal cancer. Future Oncol. 2021 Aug;17(24):3151-3162. doi: 10.2217/fon-2021-0202. Epub 2021 May 17. PMID: 33993740.

Meyerhardt JA, Shi Q, Fuchs CS, Meyer J, Niedzwiecki D, Zemla T, Kumthekar P, Guthrie KA, Couture F, Kuebler P, Bendell JC, Kumar P, Lewis D, Tan B, Bertagnolli M, Grothey A, Hochster HS, Goldberg RM, Venook A, Blanke C, O'Reilly EM, Shields AF. Effect of Celecoxib vs Placebo Added to Standard Adjuvant Therapy on Disease-Free Survival Among Patients With Stage III Colon Cancer: The CALGB/SWOG 80702 (Alliance) Randomized Clinical Trial. JAMA. 2021 Apr 6;325(13):1277-1286. doi: 10.1001/jama.2021.2454. PMID: 33821899; PMCID: PMC8025124.

Andre T, Amonkar M, Norquist JM, Shiu KK, Kim TW, Jensen BV, Jensen LH, Punt CJA, Smith D, Garcia-Carbonero R, Sevilla I, De La Fouchardiere C, Rivera F, Elez E, Diaz LA Jr, Yoshino T, Van Cutsem E, Yang P, Farooqui M, Le DT. Health-related quality of life in patients with microsatellite instability-high or mismatch repair deficient metastatic colorectal cancer treated with first-line pembrolizumab versus chemotherapy (KEYNOTE-177): an open-label, randomised, phase 3 trial. Lancet Oncol. 2021 May;22(5):665-677. doi: 10.1016/S1470-2045(21)00064-4. Epub 2021 Apr 1. PMID: 33812497.

Zaniboni A, Barone CA, Banzi MC, Bergamo F, Blasi L, Bordonaro R, Bartolomeo MD, Costanzo FD, Frassineti GL, Garufi C, Giuliani F, Latiano TP, Martinelli E, Personeni N, Racca P, Tamburini E, Tonini G, Besse MG, Spione M, Falcone A. Italian results of the PRECONNECT study: safety and efficacy of trifluridine/tipiracil in metastatic colorectal cancer. Future Oncol. 2021 Jun;17(18):2315-2324. doi: 10.2217/fon-2020-1278. Epub 2021 Mar 5. PMID: 33663264.

Aranda E, Viéitez JM, Gómez-España A, Gil Calle S, Salud-Salvia A, Graña B, Garcia-Alfonso P, Rivera F, Quintero-Aldana GA, Reina-Zoilo JJ, González-Flores E, Salgado Fernández M, Guillén-Ponce C, Garcia-Carbonero R, Safont MJ, La Casta Munoa A, García-Paredes B, López López R, Sastre J, Díaz-Rubio E; Spanish Cooperative Group for the Treatment of Digestive Tumors (TTD). FOLFOXIRI plus bevacizumab versus FOLFOX plus bevacizumab for patients with metastatic colorectal cancer and ≥3 circulating tumour cells: the randomised phase III VISNÚ-1 trial. ESMO Open. 2020 Nov;5(6):e000944. doi: 10.1136/esmoopen-2020-000944. PMID: 33148620; PMCID: PMC7640586.

Cremolini C, Antoniotti C, Rossini D, Lonardi S, Loupakis F, Pietrantonio F, Bordonaro R, Latiano TP, Tamburini E, Santini D, Passardi A, Marmorino F, Grande R, Aprile G, Zaniboni A, Murgioni S, Granetto C, Buonadonna A, Moretto R, Corallo S, Cordio S, Antonuzzo L, Tomasello G, Masi G, Ronzoni M, Di Donato S, Carlomagno C, Clavarezza M, Ritorto G, Mambrini A, Roselli M, Cupini S, Mammoliti S, Fenocchio E, Corgna E, Zagonel V, Fontanini G, Ugolini C, Boni L,

Falcone A; GONO Foundation Investigators. Upfront FOLFOXIRI plus bevacizumab and reintroduction after progression versus mFOLFOX6 plus bevacizumab followed by FOLFIRI plus bevacizumab in the treatment of patients with metastatic colorectal cancer (TRIBE2): a multicentre, open-label, phase 3, randomised, controlled trial. Lancet Oncol. 2020 Apr;21(4):497-507. doi: 10.1016/S1470-2045(19)30862-9. Epub 2020 Mar 9. PMID: 32164906.

Bridgewater JA, Pugh SA, Maishman T, Eminton Z, Mellor J, Whitehead A, Stanton L, Radford M, Corkhill A, Griffiths GO, Falk S, Valle JW, O'Reilly D, Siriwardena AK, Hornbuckle J, Rees M, Iveson TJ, Hickish T, Garden OJ, Cunningham D, Maughan TS, Primrose JN; New EPOC investigators. Systemic chemotherapy with or without cetuximab in patients with resectable colorectal liver metastasis (New EPOC): long-term results of a multicentre, randomised, controlled, phase 3 trial. Lancet Oncol. 2020 Mar;21(3):398-411. doi: 10.1016/S1470-2045(19)30798-3. Epub 2020 Jan 31. PMID: 32014119; PMCID: PMC7052737.

André T, Shiu KK, Kim TW, Jensen BV, Jensen LH, Punt C, Smith D, Garcia-Carbonero R, Benavides M, Gibbs P, de la Fouchardiere C, Rivera F, Elez E, Bendell J, Le DT, Yoshino T, Van Cutsem E, Yang P, Farooqui MZH, Marinello P, Diaz LA Jr; KEYNOTE-177 Investigators. Pembrolizumab in Microsatellite-Instability-High Advanced Colorectal Cancer. N Engl J Med. 2020 Dec 3;383(23):2207-2218. doi: 10.1056/NEJMoa2017699.
PMID: 33264544.

Kopetz S, Grothey A, Yaeger R, Van Cutsem E, Desai J, Yoshino T, Wasan H, Ciardiello F, Loupakis F, Hong YS, Steeghs N, Guren TK, Arkenau HT, Garcia-Alfonso P, Pfeiffer P, Orlov S, Lonardi S, Elez E, Kim TW, Schellens JHM, Guo C, Krishnan A, Dekervel J, Morris V, Calvo Ferrandiz A, Tarpgaard LS, Braun M, Gollerkeri A, Keir C, Maharry K, Pickard M, Christy-Bittel J, Anderson L, Sandor V, Tabernero J. Encorafenib, Binimetinib, and Cetuximab in <i>BRAF</i> V600E-Mutated Colorectal Cancer. N Engl J Med. 2019 Oct 24;381(17):1632-1643. doi: 10.1056/NEJMoa1908075. Epub 2019 Sep 30. PMID: 31566309.

Van Cutsem E, Huijberts S, Grothey A, Yaeger R, Cuyle PJ, Elez E, Fakih M, Montagut C, Peeters M, Yoshino T, Wasan H, Desai J, Ciardiello F, Gollerkeri A, Christy-Bittel J, Maharry K, Sandor V, Schellens JHM, Kopetz S, Tabernero J. Binimetinib, Encorafenib, and Cetuximab Triplet Therapy for Patients With <i>BRAF</i> V600E-Mutant Metastatic Colorectal Cancer: Safety Lead-In Results From the Phase III BEACON Colorectal Cancer Study. J Clin Oncol. 2019 Jun 10;37(17):1460-1469. doi: 10.1200/JCO.18.02459. Epub 2019 Mar 20. PMID: 30892987; PMCID: PMC7370699.

Kwakman JJM, van Werkhoven E, Simkens LHJ, van Rooijen JM, van de Wouw YAJ, Tije AJT, Creemers GM, Hendriks MP, Los M, van Alphen RJ, Polée MB, Muller EW, van der Velden AMT, van Voorthuizen T, Koopman M, Mol L, Punt CJA. Updated Survival Analysis of the Randomized Phase III Trial of S-1 Versus Capecitabine in the First-Line Treatment of Metastatic Colorectal Cancer by the Dutch Colorectal Cancer Group. Clin Colorectal Cancer. 2019 Jun;18(2):e229-e230. doi: 10.1016/j.clcc.2019.01.002. Epub 2019 Jan 29. PMID: 30782413.

Qin S, Li J, Wang L, Xu J, Cheng Y, Bai Y, Li W, Xu N, Lin LZ, Wu Q, Li Y, Yang J, Pan H, Ouyang X, Qiu W, Wu K, Xiong J, Dai G, Liang H, Hu C, Zhang J, Tao M, Yao Q, Wang J, Chen J, Eggleton SP, Liu T. Efficacy and Tolerability of First-Line Cetuximab Plus Leucovorin, Fluorouracil, and Oxaliplatin (FOLFOX-4) Versus FOLFOX-4 in Patients With <i>RAS</i> Wild-Type Metastatic Colorectal Cancer: The Open-Label, Randomized, Phase III TAILOR Trial. J Clin Oncol. 2018 Oct 20;36(30):3031-3039. doi: 10.1200/JCO.2018.78.3183. Epub 2018 Sep 10. PMID: 30199311; PMCID: PMC6324088.

Li J, Xu R, Qin S, Liu T, Pan H, Xu J, Bi F, Lim R, Zhang S, Ba Y, Bai Y, Fan N, Tsuji A, Yeh KH, Ma B, Wei V, Shi D, Magherini E, Shen L. Aflibercept plus FOLFIRI in Asian patients with pretreated metastatic colorectal cancer: a randomized Phase III study. Future Oncol. 2018 Aug;14(20):2031-2044. doi: 10.2217/fon-2017-0669. Epub 2018 Aug 17. Erratum in: Future Oncol. 2019 Feb;15(4):451. PMID: 30117334.

Pinto C, Normanno N, Orlandi A, Fenizia F, Damato A, Maiello E, Tamburini E, Di Costanzo F, Tonini G, Bilancia D, Corsi D, Pisconti S, Ferrau F, Gori S, Daniele B, Zaniboni A, Soto Parra H, Frassinetti GL, Iaffaioli RV, Cassata A, Zampino MG, Repetto L, Calegari MA, Barone C; all the investigators of ERMES study group.. Phase III study with FOLFIRI + cetuximab versus FOLFIRI + cetuximab followed by cetuximab alone in RAS and BRAF WT mCRC. Future Oncol. 2018 Jun;14(14):1339-1346. doi: 10.2217/fon-2017-0592. Epub 2018 May 30. PMID: 29846100.

Yamada Y, Denda T, Gamoh M, Iwanaga I, Yuki S, Shimodaira H, Nakamura M, Yamaguchi T, Ohori H, Kobayashi K, Tsuda M, Kobayashi Y, Miyamoto Y, Kotake M, Shimada K, Sato A, Morita S, Takahashi S, Komatsu Y, Ishioka C. S-1 and irinotecan plus bevacizumab versus mFOLFOX6 or CapeOX plus bevacizumab as first-line treatment in patients with metastatic colorectal cancer (TRICOLORE): a randomized, open-label, phase III, noninferiority trial. Ann Oncol. 2018 Mar 1;29(3):624-631. doi: 10.1093/annonc/mdx816. PMID: 29293874; PMCID: PMC5889030.

Adams R, Brown E, Brown L, Butler R, Falk S, Fisher D, Kaplan R, Quirke P, Richman S, Samuel L, Seligmann J, Seymour M, Shiu KK, Wasan H, Wilson R, Maughan

T; FOCUS4 Trial Investigators. Inhibition of EGFR, HER2, and HER3 signalling in patients with colorectal cancer wild-type for BRAF, PIK3CA, KRAS, and NRAS (FOCUS4-D): a phase 2-3 randomised trial. Lancet Gastroenterol Hepatol. 2018 Mar;3(3):162-171. doi: 10.1016/S2468-1253(17)30394-1. Epub 2017 Dec 16. PMID: 29254887; PMCID: PMC6125825.

Cascinu S, Rosati G, Nasti G, Lonardi S, Zaniboni A, Marchetti P, Leone F, Bilancia D, Iaffaioli RV, Zagonel V, Giordano M, Corsi DC, Ferraú F, Labianca R, Ronzoni M, Scartozzi M, Galli F; GISCAD investigators. Treatment sequence with either irinotecan/cetuximab followed by FOLFOX-4 or the reverse strategy in metastatic colorectal cancer patients progressing after first-line FOLFIRI/bevacizumab: An Italian Group for the Study of Gastrointestinal Cancer phase III, randomised trial comparing two sequences of therapy in colorectal metastatic patients. Eur J Cancer. 2017 Sep;83:106-115. doi: 10.1016/j.ejca.2017.06.029. Epub 2017 Jul 20. PMID: 28735067.

Cremolini C, Marmorino F, Loupakis F, Masi G, Antoniotti C, Salvatore L, Schirripa M, Boni L, Zagonel V, Lonardi S, Aprile G, Tamburini E, Ricci V, Ronzoni M, Pietrantonio F, Valsuani C, Tomasello G, Passardi A, Allegrini G, Di Donato S, Santini D, Falcone A; all the investigators of the Gruppo Oncologico del Nord Ovest. TRIBE-2: a phase III, randomized, open-label, strategy trial in unresectable metastatic colorectal cancer patients by the GONO group. BMC Cancer. 2017 Jun 9;17(1):408. doi: 10.1186/s12885-017-3360-z. PMID: 28599628; PMCID: PMC5466800.

Guren TK, Thomsen M, Kure EH, Sorbye H, Glimelius B, Pfeiffer P, Österlund P, Sigurdsson F, Lothe IMB, Dalsgaard AM, Skovlund E, Christoffersen T, Tveit KM. Cetuximab in treatment of metastatic colorectal cancer: final survival analyses and extended RAS data from the NORDIC-VII study. Br J Cancer. 2017 May 9;116(10):1271-1278.
doi: 10.1038/bjc.2017.93. Epub 2017 Apr 11. PMID: 28399112; PMCID: PMC5482736.

Kwakman JJM, Simkens LHJ, van Rooijen JM, van de Wouw AJ, Ten Tije AJ, Creemers GJM, Hendriks MP, Los M, van Alphen RJ, Polée MB, Muller EW, van der Velden AMT, van Voorthuizen T, Koopman M, Mol L, van Werkhoven E, Punt CJA. Randomized phase III trial of S-1 versus capecitabine in the first-line treatment of metastatic colorectal cancer: SALTO study by the Dutch Colorectal Cancer Group. Ann Oncol. 2017 Jun 1;28(6):1288-1293. doi: 10.1093/annonc/mdx122. PMID: 28383633.

Yamazaki K, Nagase M, Tamagawa H, Ueda S, Tamura T, Murata K, Eguchi Nakajima T, Baba E, Tsuda M, Moriwaki T, Esaki T, Tsuji Y, Muro K, Taira K, Denda T, Funai S, Shinozaki K, Yamashita H, Sugimoto N, Okuno T, Nishina T,

Umeki M, Kurimoto T, Takayama T, Tsuji A, Yoshida M, Hosokawa A, Shibata Y, Suyama K, Okabe M, Suzuki K, Seki N, Kawakami K, Sato M, Fujikawa K, Hirashima T, Shimura T, Taku K, Otsuji T, Tamura F, Shinozaki E, Nakashima K, Hara H, Tsushima T, Ando M, Morita S, Boku N, Hyodo I. Randomized phase III study of bevacizumab plus FOLFIRI and bevacizumab plus mFOLFOX6 as first-line treatment for patients with metastatic colorectal cancer (WJOG4407G). Ann Oncol. 2016 Aug;27(8):1539-46. doi: 10.1093/annonc/mdw206. Epub 2016 May 13. PMID: 27177863.

Aparicio T, Lavau-Denes S, Phelip JM, Maillard E, Jouve JL, Gargot D, Gasmi M, Locher C, Adhoute X, Michel P, Khemissa F, Lecomte T, Provençal J, Breysacher G, Legoux JL, Lepère C, Charneau J, Cretin J, Chone L, Azzedine A, Bouché O, Sobhani I, Bedenne L, Mitry E; FFCD investigators. Randomized phase III trial in elderly patients comparing LV5FU2 with or without irinotecan for first-line treatment of metastatic colorectal cancer (FFCD 2001-02). Ann Oncol. 2016 Jan;27(1):121-7. doi: 10.1093/annonc/mdv491. Epub 2015 Oct 20. PMID: 26487578.
