# Supplementary material for: Tumour Burden Reporting in Phase III Clinical Trials of Metastatic Lung, Breast, and Colorectal Cancers: A Systematic Review
Source: Cancers (Basel). 2022 Jul 3;14(13):3262. doi: 10.3390/cancers14133262 (PMC9264965; doi:10.3390/cancers14133262)
Supplement: Supplementary file 1 [file cancers-14-03262-s001.zip › Supplementary File 3.pdf]

Supplementary File 3. Description of study identifiers, treatment arms and outcome-related variables.

Year of publication	First author	Study acronym or identifier	No. of pts	Arm 1	Arm 2	Arm 3	Primary end-point	HR	95% CI		*P*	Study conclusion
**Lung cancer**												
2016	Quoix E	TIME	222	Placebo+CT	TG4010+CT	-	PFS	0.74	0.55	0.98	0.019	Positive
2016	Reck M	KEYNOTE-024	305	CT	Pemb	-	PFS	0.50	0.37	0.68	<0.001	Positive
2017	Edelman MJ	CALGB 30801	312	Placebo+CT	Celecoxib+CT	-	PFS	1.07	0.85	1.36	0.3862	Negative
2017	Ferry D	BTOG2 trial	1363	GEM+CIS80[A]	GEM+CIS50	GEM+Carbo[B]	OS	0.93	NR	1.04	NR	Non inferior
2017	Govindan R	CA184104	749	Placebo+CT	Ipili+CT	-	OS	0.91	0.77	1.07	0.25	Negative
2017	Hida T	J-ALEX	207	Alectinib	Crizotinib	-	PFS	0.34	0.17	0.71	<0.0001	Positive
2017	Mok TSK	AURA3	419	Pem+Platinum	Osimertinib	-	PFS	0.30	0.23	0.41	<0.001	Positive
2017	Park CK	TRAIL	148	CIS+Peme	CIS+DTX	-	PFS	NR	NR	NR	NR	Non inferior
2017	Shi YK	CONVINCE	285	CIS+Peme	Icotinib	-	PFS	0.61	0.43	0.87	0.006	Positive
2017	Soria JC	ASCEND-4	376	Platinum-based CT	Ceritinib	-	PFS	0.55	0.42	0.73	<0.00001	Positive
2017	Wu YL	ARCHER 1050	452	Gef	Dacomitinib	-	PFS	0.59	0.47	0.74	<0.0001	Positive
2018	Gandhi L	KEYNOTE-189	616	Placebo+Platinum+ Peme	Pemb+Platinum+ Peme	-	PFS/OS	0.49	0.38	0.64	<0.001	Positive
2018	Herbst RS	SWOG S0819	1313	Carbo+PTX w/o Beva	Carbo+PTX+Cet w/o Beva	-	PFS/OS	0.93	0.83	1.04	0.22	Negative
2018	Ouyang X	NCT03083743	452	Placebo+VNR+CIS	Dulanermin+VNR +CIS	-	PFS	0.40	0.31	0.51	< 0.0001	Positive
2018	Paz-Ares L	KEYNOTE-407	559	Placebo+CT	Pemb+CT	-	PFS/OS	0.64	0.49	0.85	<0.001	Positive
2018	Socinski MA	IMpower150	1202	Atezo+Carbo+ PTX[A]	Atezo+Carbo+ PTX+Beva[A]	Carbo+PTX+ Beva[B]	PFS/OS	0.78	0.64	0.96	0.02	Positive
2019	Camidge DR	ALEX	303	Alectinib	Crizotinib	-	PFS	0.43	0.32	0.58	NR	Positive
2019	Hellmann MD	CheckMate 227	1189	Nivo+CT[A]	Nivo+Ipili	CT[B]	OS	0.79	0.65	0.96	0.007	Positive
2019	Kelly RJ	NCT02588261	530	Erlotinib[A]	ASP8273[B]	Gef[A]	PFS	1.61	1.08	2.39	0.992	Negative
2019	Mok TSK	KEYNOTE-042	1274	CT	Pemb	-	OS	0.69	0.56	0.85	0.0003	Positive
2019	Reck M	KEYNOTE-024	305	CT	Pemb	-	OS	0.63	0.47	0.86	0.002	Positive
2019	West H	IMpower130	723	CT	Atezo+CT	-	PFS/OS	0.79	0.64	0.98	0.033	Positive
2019	Yoshioka H	WJTOG 3405	172	CT	Gef	-	OS	1.25	0.88	1.77	0.2070	Negative
2019	Zhou C	ALESIA	187	Crizotinib	Alectinib	-	PFS	0.22	0.13	0.38	<0.0001	Positive
2020	Jotte R	IMpower131	1021	Atezo+Carbo+ PTX[A]	Atezo+Carbo+ NAB-PTX[A]	Carbo+ NAB-PTX[B]	PFS	0.71	0.60	0.85	0.0001	Positive
2020	Herbst RS	IMpower110	572	CT	Atezo	-	OS	0.59	0.40	0.89	<0.01	Positive

2020	Hosomi Y	NEJ009	345	Gef	Gef+Carbo+Peme	-	PFS/OS	0.72	0.55	0.95	0.021	Positive
2020	Noronha V	CTRI/2016/08/ 007149	350	Gef	Gef+Peme+Carbo	-	PFS	0.51	0.39	0.66	0.001	Positive
2020	Okamoto I	UMIN 000011460	433	Carbo+Peme	DTX	-	OS	0.85	0.68	1.05	0.003	Non inferior
2020	Peters S	SPLENDOUR	509	CT	CT+denosumab	-	OS	0.96	0.78	1.19	0.36	Negative
2020	Ramalingam SS	Flaura	556	Standard EGFR-TKI	Osimertinib	-	OS	0.8	0.64	1.00	0.046	Positive
2020	Rizvi NA	MYSTIC	1118	CT[A]	Durv[B]	Durv+Treme[B]	PFS/OS	0.76	0.56	1.02	0.04	Negative
2020	Shaw AT	CROWN	296	Crizotinib	Lorlatinib	-	PFS	0.28	0.19	0.41	<0.001	Positive
2020	Yang Y	ORIENT-11	397	Placebo+Platinum+ Peme	Sintilimab+ Platinum+Peme	-	PFS	0.48	0.36	0.64	<0.00001	Positive
2021	Lu S	RATIONALE 304	332	Platinum+Peme	Platinum+Peme+ tislelizumab	-	PFS	0.64	0.46	0.90	0.0044	Positive
2021	Nishio M	IMpower132	578	Platinum+Peme	Atezo+Platinum+ Peme	-	PFS/OS	0.81	0.64	1.03	0.0797	Negative
2021	Paz-Ares L	CheckMate 9LA	719	CT	Nivo+Ipili+CT	-	OS	0.69	0.55	0.87	0.00065	Positive
2021	Ramalingam SS	NCT02106546	970	Placebo+Carbo+PTX	Veliparib+Carbo+ PTX	-	OS	0.90	0.74	0.97	0.266	Negative
2021	Rodríguez-Abreu D	KEYNOTE-189	616	Placebo+CT	Pemb+CT	-	PFS/OS	0.56	0.46	0.69	NR	Positive
2021	Sezer A	EMPOWER-Lung 1	563	CT	Cemiplimab	-	PFS/OS	0.57	0.42	0.77	0.0002	Positive
2021	Wang J	RATIONALE-307	355	Tis+Carbo+PTX[A]	Tis+Carbo+ NAB-PTX[A]	Carbo+PTX[B]	PFS	0.52	0.37	0.74	<0.001	Positive
2021	Zhou C	CameL	412	Carbo+Peme	Camrel+ Carbo+Peme	-	PFS	0.6	0.45	0.79	0.0001	Positive
2021	Zhou C	ORIENT-12	357	Placebo+CIS+GEM	Sintilimab+CIS+ GEM	-	PFS	0.53	0.42	0.68	<0.00001	Positive
**Breast cancer**												
2016	Martin M	BELLE-4	416	Placebo+PTX	Buparlisib+PTX	-	PFS	1.18	0.82	1.68	NR	Negative
2016	Miles D	MERiDiAN	481	Placebo+PTX	Beva+PTX	-	PFS	0.68	0.53	0.88	0.0038	Positive
2016	Welt, A	CARIN	600	Cape+Beva	Cape+Beva+VNR		PFS	0.84	0.7	1.1	0.058	Negative
2016	Zielinski C	TURANDOT	531	Beva+Cape	Beva+PTX	-	OS	1.02	0.99	1.26	0.0070	Positive
2017	Harbeck N	PELICAN	210	Cape	Pegylated liposomal DOXO	-	PFS	1.21	0.83	1.75	0.64	Negative

2017	Perez EA	MARIANNE	1095	Placebo+ TDM-1[A]	Taxanes+Tra[B]	Pert+ TDM-1[A]	PFS	0.91	0.73	1.13	0.31	Non inferior
2017	Von Minckwitz G	KATHERINE	1486	TDM-1	Trastuzumab	-	PFS	0.50	0.39	0.64	<0.000032	Positive
2018	Takashima T	SELECT-BC	618	Taxanes	S-1	-	PFS	1.05	0.86	1.27	0.05	Non inferior
2018	Yardley DA	tnAcity	191	NAB-PTX+GEM[A]	NAB-PTX+Carbo[A]	Carbo+GEM[B]	PFS	0.59	0.38	0.92	0.02	Positive
2019	Hortobagyi GN	MONALEESA -2	668	Placebo+ Letrozole	Ribociclib+ Letrozole	-	PFS	0.56	0.45	0.70	0.0000000 963	Positive
2019	Rugo HS	PALOMA-2	666	Placebo+ Letrozole	Palbociclib+ Letrozole	-	PFS	0.56	0.46	0.68	<0.0001	Positive
2020	Cortes J	KEYNOTE-355	847	Placebo+CT	Pemb+CT	-	PFS/OS	0.82	0.69	0.97	NR	Positive
2020	Schmid P	IMpassion130	902	Placebo+NAB-PTX	Atezo+NAB-PTX	-	PFS/OS	0.86	0.72	1.02	0.078	Negative
2020	Slamon DJ	MONALEESA -3	726	Placebo+ Fulvestrant	Ribociclib+ Fulvestrant	-	PFS	0.55	0.42	0.72	NR	Positive
2020	Swain SM	CLEOPATRA	808	Placebo+DTX+Tra	Pert+DTX+Tra	-	PFS	0.69	0.58	0.81	<0.001	Positive
2021	Goetz MGP	Monarch 3	493	Placebo+NSAI	Abemaciclib+ NSAI	-	PFS	0.54	0.41	0.72	0.000021	Positive
2021	Miles D	IMpassion131	651	Placebo+PTX	Atezo+PTX	-	PFS	0.82	0.60	1.12	0.20	Negative
**Colorectal cancer**												
2016	Aparicio T	FFCD 2001-02	282	FU	Folfiri	-	PFS	0.84	0.66	1.07	0.15	Negative
2016	Yamazaki K	WJOG4407G	395	Folfox+Beva	Folfiri+Beva	-	PFS	0.90	0.72	1.13	0.003	Non inferior
2017	Guren TK	NORDIC-VII	566	FLOX[A]	FLOX+Cet[B]	FLOX int+Cet[B]	PFS	0.89	0.72	1,11	0.31	Negative
2018	Adams R	FOCUS4-D	32	Placebo	AZD8931	-	PFS	1.10	0.47	3.57	0.95	Negative
2018	Qin S	TAILOR	393	Folfox	Fofox+Cet	-	PFS	0.69	0.54	0.89	0.004	Positive
2018	Yamada Y	TRICOLORE	484	FU+Oxa+Beva	S-1+Iri+Beva	-	PFS	0.84	0.70	1.02	<0.0001	Non inferior
2020	André T	KEYNOTE-177	307	CT	Pemb	-	PFS/OS*	0.60	0.45	0.80	0.0002	Positive
2020	Aranda E	VISNÚ-1	349	Fofloxiri+Beva	Folfox+Beva	-	PFS	0.64	0.49	0.82	0.0006	Positive
2020	Cremolini C	TRIBE-2	679	Folfoxiri+Beva→ Folfoxiri+ Beva	Folfox+Beva→ Folfiri+Beva	-	PFS	0.74	0.63	0.88	0.0005	Positive
2021	Qin S	NCT03511963	677	FU+Oxa+Beva	FU+Oxa+HLX04	-	PFS	0.92	0.80	1.05	NR	Equivalent

A total of 70 studies were analyzed.

Atezo: atezolizumab; Beva: bevacizumab; Camrel: Camrelizumab; Cape: capecitabine; Carbo: carboplatin; Cet: cetixumab; CI: confidence intervals; CIS: cisplatin; CT: standard chemotherapy (standard schedule bewteen multiple available options at Oncolgist discretion); DOXO: doxorubicin; DTX: docetaxel; FOLFIRI: 5-fluorouracil + leucovorin + irinotecan chemotherapy; FLOX and FOLFOX: 5-fluorouracil + leucovorin + oxaliplatin chemotherapy; FOLFOXIRI: 5-fluorouracil + leucovorin + oxaliplatin + irinotecan chemotherapy; Gef: gefitinib; GEM: gemcitabine; HR: hazard ratio; int: intermittent; Ipili: ipilimumab; NAB-PTX: nab-paclitaxel; Nivo: nivolumab; NSAI: nonsteroidal aromatase inhibitor; OS: overall survival; Pemb: pembrolizumab; Peme: pemetrexed; Pert: pertuzumab: PFS: progression-

free survival; pm-PTX: polymeric micellar-Paclitaxel; PTL: primary tumour location; PTX: paclitaxel; TDM-1: TDM-1:trastuzumab emtansine; Tra: trastuzuamb; Tis: Tislelizumab; Treme: tremelimumab; VNR: Vinorelbine. W/o: with or without.

When PFS and OS are co-primary end-points, the reported HRs refer to OS.

*Here, HR refers to PFS (HR for OS was not reported).

[A] vs [B]: dichotomization for reported HR.
