# Supplementary material for: Tumour Burden Reporting in Phase III Clinical Trials of Metastatic Lung, Breast, and Colorectal Cancers: A Systematic Review
Source: Cancers (Basel). 2022 Jul 3;14(13):3262. doi: 10.3390/cancers14133262 (PMC9264965; doi:10.3390/cancers14133262)
Supplement: Supplementary file 1 [file cancers-14-03262-s001.zip › Supplementary File 4.pdf]

Supplementary File 4. Description of disease-extent related variables in the selected studies.

PTL	Study acronym or identifier	No. of pts	No. of pts with LBD	Definition of LBD	Disease extent as stratification factor	Description of oligo-metastatic disease	Subgroup analysis according to LBD or no. of metastatic sites	No of pts with LBD Arm 1	No of pts with LBD Arm 2	No of pts with LBD Arm 3
**Lung**	TIME	222	-	No	No	No	No	-	-	-
	KEYNOTE-024	305	-	No	No	No	No	-	-	-
	CALGB 30801	312	-	No	Yes (stage III *vs* IV)	No	No	-	-	-
	BTOG2 trial	1363	-	No	Yes (stage III *vs* IV)	No	No	-	-	-
	CA184104	749	-	No	No	No	No	-	-	-
	J-ALEX	207	-	No	Yes (stage III *vs* IV)	No	No	-	-	-
	AURA3	419	-	No	No	No	No	-	-	-
	TRAIL	148	-	No	No	No	No	-	-	-
	CONVINCE	285	183	1 met site	No	No	Yes	-	-	-
	ASCEND-4	376	177	<median SOD for target lesions	No	No	No	-	-	-
	ARCHER 1050	452	-	No	No	No	No	-	-	-
	KEYNOTE-189	616	-	No	No	No	No	-	-	-
	SWOG S0819	1313	-	No	Yes (M1a vs M1b)	No	No	-	-	-
	NCT03083743	452	-	No	Yes (stage III *vs* IV)	No	No	74/342 (21.6%)	24/110 (21.8%)	-
	KEYNOTE-407	559	-	No	No	No	No	-	-	-
	IMpower150	1202	-	No	Yes liver met (yes vs no)	No	No	-	-	-
	ALEX	303	-	No	Yes liver met (yes vs no)	No	No	-	-	-
	CheckMate 227	1189	-	No	No	No	No	-	-	-
	NCT02588261	530	-	No	No	No	No	-	-	-
	KEYNOTE-042	1274	-	No	No	No	No	-	-	-
	KEYNOTE-024	305	-	No	No	No	No	-	-	-
	IMpower130	723	-	No	Yes	No	No	-	-	-

					(stage III *vs* IV)					
	WJTOG 3405	172	-	No	No	No	No	-	-	-
	ALESIA	187	-	No	No	No	No	-	-	-
	IMpower131	1021	-	No	Yes CNS met (yes vs no)	No	No	-	-	-
	IMpower110	572	-	No	No	No	No	-	-	-
	NEJ009	345	-	No	Yes (stage III *vs* IV)	No	No	-	-	-
	CTRI/2016/08/007149	350	-	No	No	No	No	-	-	-
	UMIN 000011460	433	-	No	Yes (stage III *vs* IV)	No	No	-	-	-
	SPLENDOUR	509	-	No	No	No	No	-	-	-
	Flaura	556	-	No	No	No	No	-	-	-
	MYSTIC	1118	-	No	No	No	No	-	-	-
	CROWN	296	-	No	No	No	No	-	-	-
	ORIENT-11	397	-	No	No	No	No	-	-	-
	RATIONALE 304	334	-	No	Yes (stage III *vs* IV)	No	No	-	-	-
	IMpower132	578	-	No	No	No	No	-	-	-
	CheckMate 9LA	719	-	No	No	No	No	-	-	-
	NCT02106546	970	608	1-2 met sites	Yes (stage III *vs* IV)	No	No	310 (64.0%)	298 (61.3%)	-
	KEYNOTE-189	616	-	No	No	No	No	-	-	-
	EMPOWER-Lung 1	563	-	No	No	No	No	-	-	-
	RATIONALE-307	355	-	No	Yes (stage III *vs* IV)	No	No	-	-	-
	CameL	412	-	No	No	No	No	-	-	-
	ORIENT-12	357	-	No	Yes	No	No	-	-	-
**Breast**	BELLE 4	416	-	-	No	No	No	-	-	-
	MERiDiAN	481	229	<3 met sites	No	No	Yes	117/242 (48.3%)	112/239 (46.8%)	-
	CARIN	600	408	< 3 met sites	No	No	Yes	206/297 (69.4%)	202/295 (68.5%)	-
	TURANDOT*	531	144	1 met site	No	No	Yes	58/265 (21.8%)	86/266 (32.3%)	-
	PELICAN	210	-	No	No	No	No	-	-	-
	MARIANNE	1095	-	No	No	No	No	-	-	-
	KATHERINE	1486	-	No	No	No	No	-	-	-

	SELECT BC	618	-	No	No	No	No	-	-	-
	tnAcity	191	-	No	No	No	No	-	-	-
	MONALEESA-2	668	217	1 met site	No	No	No	117/334 (35.0%)	100/334 (29.9%)	-
	PALOMA-2	666	204	1 met site	No	No	Yes	66/222 (29.7%)	138/444 (31.1%)	-
	KEYNOTE-355	847	479	< 3 met sites	No	No	Yes	166/281 (59.1%)	313/566 (55.3%)	-
	IMpassion130	902	673	<4 met sites	No	No	Yes	341/451 (75.6%)	332/451 (73.6%)	-
	MONALEESA-3	726	455	<3 met sites	No	No	Yes	147/242 (60.7%)	308/484 (63.6%)	-
	CLEOPATRA	806	-	No	No	No	No	-	-	-
	MONARCH 3	493	-	No	No	No	No	-	-	-
	IMpassion131*	651	412	<4 met sites	Yes liver met (yes vs no)	No	Yes	172/220 (78.2%)	240/431 (55.7%)	-
**Colon**	FFCD 2001-02	282	120	1 met site	No	No	Yes	62/142 (43.7)	58/138 (42.0)	-
	WJOG4407G	395	177	1 met site	Yes liver met (yes vs no)	No	No	86/198 (43.4%)	91/197 (46.2%)	-
	NORDIC-VII	566	203	1 met site	No	No	No	76/194 (39.2%)	68/185 (36.7%)	59/187 (31.5%)
	FOCUS4-D	32	15	1 met site	No	No	No	6/16 (37.5%)	9/16 (56.2%)	-
	TAILOR	393	152	1 met site	No	No	Yes	80/200 (40.0%)	72/193 (37.3%)	-
	TRICOLORE	484	251	1 met site	Yes (1 *vs* >1 met site)	No	Yes	124/243 (51.0%)	127/241 (52.7%)	-
	KEYNOTE-177	307	-	No	No	No	No	-	-	-
	VISNÚ-1	349	133	1 met site	Yes (1 *vs* >1 met site)	No	Yes	65/177 (36.7%)	68/172 (39.5%)	-
	TRIBE-2	679	278	1 met site	No	No	No	127/340 (37.3%)	151/339 (44.5%)	-
	NCT03511963	677	-	No	No	No	No	-	-	-

**P* significant at χ2 test for unbalances in number of patients (low-burden vs high-burden diseases) exposed to different treatment arms.

LBD: low-burden disease; met: metastatic; patients: pts; PTL: primary tumour location; SOD: sum of diameter.
