# Supplementary material for: Tumour Burden Reporting in Phase III Clinical Trials of Metastatic Lung, Breast, and Colorectal Cancers: A Systematic Review
Source: Cancers (Basel). 2022 Jul 3;14(13):3262. doi: 10.3390/cancers14133262 (PMC9264965; doi:10.3390/cancers14133262)
Supplement: Supplementary file 1 [file cancers-14-03262-s001.zip › Supplementary File 5.pdf]

Supplementary File 5

Effect of oligo-metastatic colorectal cancer (omCRC) patients on the prognosis of a cohort with clinical characteristics permissive for inclusion in phase III trials.

Background

We depicted and analysed survival of 112 consecutive mCRC patients who had, at treatment start, clinical characteristics consistent with those of inclusion criteria of phase III studies and, therefore, potentially eligible to be enrolled in clinical trials. Furthermore, survival was differentiated according to disease extent (poly- vs oligo-metastatic disease) at start of first-line therapy.

Methods

A clinical cohort of metastatic colorectal cancer (mCRC) patients treated at the Department of Abdominal Oncology, sub-structure of Innovative Therapies for Abdominal Cancers, Istituto Nazionale Tumouri (National Cancer Institute), "G. Pascale" foundation, was described and analysed. Data collection was performed from an institutional electronic database prospectively updated and it was limited to patients starting first-line chemotherapy during the last three years (January 2019-December 2021). All patients included had PS (Performance Status) ECOG (Eastern Cooperative Oncology Group) 0-1, no brain metastases, age<75 years, no decompensated comorbidities, life expectancy>3 months. Oligo-metastatic disease was defined as cancer involving one to three lesions per organ with a maximum tumour diameter smaller than 7 cm.
Overall survival (OS) was measured from the start of the first-line chemotherapy until death from any cause and it was depicted through the Kaplan-Meier curves. The Log-Rank test was used to evaluate the statistical significance of divergent survivals according to the extent of disease at start of first-line chemotherapy. Cox proportional hazards regression model was applied to analyse the effect of potential predictors (covariates) of OS in the clinical cohort of mCRC patients. Covariates (age, gender, side of primary tumour, response to first-line therapy, RAS/BRAF gene status, oligo-metastatic disease) were selected after consensus discussion between authors and were dichotomized. The reported HR (see Results) is the instantaneous relative risk of death, at any time, for an individual with the risk factor present compared with an individual with the risk factor absent, given both individuals are the same on all other covariates. 95% confidence intervals (CI) of HR are also reported. According to our internal policies, the institutional review board approval was not required for the retrospective analysis of this clinical cohort.

Results

Median OS of the entire cohort (mCRC patients) was 24.0 months (95% CI: 22.2-24.8). Interestingly, there was an evident and statistically significant difference between pmCRC (poly-metastatic colorectal cancer) and omCRC patients in terms of survival (median OS for omCRC: not reached; median OS for pmCRC: 22.0 months; P=0.0006 at Log Rank test). In a Cox proportional hazards regression model to analyse the effect of potential predictors of OS in this clinical series (male vs female, right vs left side of primary tumour, response or not to first-line therapy, wild-type vs mutated RAS/BRAF, <65 years vs >65 years age, oligo- vs poly-metastatic disease), the oligo-metastatic status emerged as a strong and independent variable at treatment start (HR: 0.17; 95% CI: 0.05-0.56; P=0.0034).

Some patients were indeed enrolled in clinical trials (see symbols beside the curves used for anonymized identification of the clinical trials in **Supplementary Figure 1**): 23 out of 98 pmCRC (23.5%), and 7 out of 14 omCRC patients (50.0%). In particular, the median survival of the entire/undifferentiated mCRC cohort (including also omCRC patients) is 24.0 months compared to 22.0 months of true pmCRC patients (excluding omCRC patients). Therefore, the contamination of omCRC patients improves the prognosis (+2.0 months).

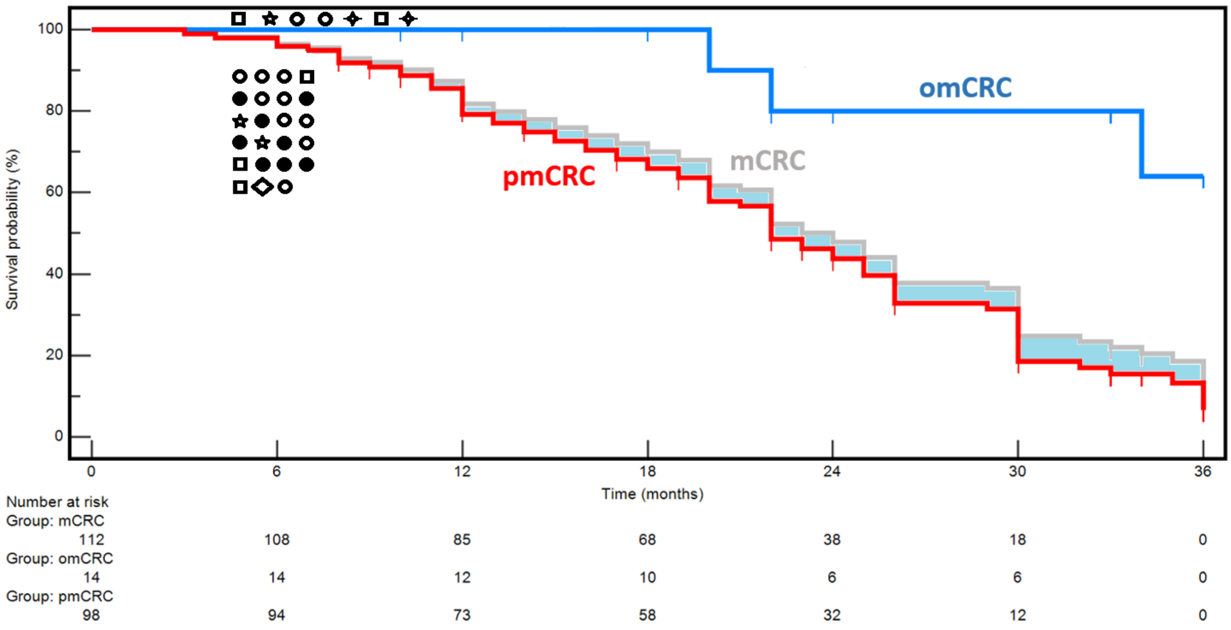

Supplementary Figure 1. Survival curves of pmCRC (poly-metastatic colorectal cancer patients) red line, mCRC (all metastatic colorectal cancer patients) gray line, omCRC (oligo-metastatic colorectal cancer patients) blue line. Beside the curves, anonymized symbols identify trials in which have been enrolled some of the consecutive patients (7 omCRC and 23 pmCRC) included in our analysis. Below are reported number at risk (death) every six months. The spaces filled in blue represent the survival difference between the curve of pmCRC patient (red curve) and of all mCRC patients (pmCRC plus omCRC, grey curve).

Conclusions

This supplementary evidence suggests that the prognosis of this mCRC cohort is positively influenced by omCRC patients.
